# Design and Experimental Validation of an Embedded Edge Artificial Intelligence Camera System for Real-Time Intelligent Vision

**DOI:** 10.3390/s26206498

**Published:** 2026-10-14

**Authors:** Hsiu-Ying Hwang, Fu-Hsiang Huang

**Affiliations:** 1 Department of Vehicle Engineering, National Taipei University of Technology, No. 1, Section 3, Zhongxiao E. Rd., Taipei 106344, Taiwan; t113669002@ntut.edu.tw or davis.huang@ait-group.com; 2 Asian Information Technology Inc., Taipei 115018, Taiwan

**Keywords:** edge artificial intelligence camera, embedded system, edge computing, real-time inference, signal integrity, license plate recognition

## Abstract

This study presents the design and experimental validation of an embedded edge artificial intelligence (AI) camera system for intelligent vision applications. The developed system integrates a multicore edge computing processor and a dedicated AI engine into a compact smart camera platform, enabling on-device image processing, intelligent recognition, and localized decision-making. The hardware implementation incorporates circuit design, impedance-controlled high-speed signal routing, and printed circuit board (PCB) stack-up design. The signal integrity of the MIPI Display Serial Interface (MIPI-DSI) is evaluated through scattering-parameter simulations and measurements, while the conducted radio frequency (RF) transmitter performance and radiated emissions of the fabricated prototype are experimentally assessed. In an outdoor and indoor parking-lot field test, the integrated system processed images at approximately 2 frames per second (FPS) while operating in multi-task mode at an image resolution of 1280 × 960 pixels. When configured exclusively for license plate recognition, the system achieves a processing throughput of up to 45 FPS. For all 13 tested vehicles, comprising two buses, two trucks, and nine cars, both the predicted vehicle type and complete license plate number matched the ground truth, yielding an integrated recognition accuracy of 12/13 for this test sample. Representative measurements of the prototype’s DC input power were approximately 2.20–6.32 W at a supply voltage of 12 V. These results demonstrate the feasibility of the developed edge AI camera for fixed-location parking-management applications and real-time edge inference capability.

## 1. Introduction

Camera devices are widely used in applications such as home and community security, urban traffic management, video surveillance, vehicle safety, assisted driving, parking management systems, and many others. Conventional AI-based vision systems often rely on external PCs or cloud servers for image processing and inference, which may increase system size, communication latency, bandwidth requirements, and power consumption. To improve recognition accuracy, additional cameras, high-bandwidth network infrastructure, and increasingly complex algorithms are often required, resulting in higher system cost, greater energy consumption, and increased demand for network resources.

In recent years, advances in semiconductor fabrication processes and 3D packaging technologies have enabled next-generation processors with high computational performance and low power consumption. Consequently, single-chip edge computing processors and heterogeneous System-on-Chip (SoC) platforms for camera-based and Advanced Driver Assistance Systems (ADAS) applications were introduced. Through algorithm optimization and hardware integration, these embedded platforms can perform image acquisition, detection, and recognition tasks locally, reducing system latency and dependence on cloud computing resources. Representative examples include the Texas Instruments Jacinto TDAx family of ADAS SoCs, which supports front/rear camera systems, long-range radar (LRR), surround-view electronic control units (ECUs), ultra-short-range radar, and ultrasonic parking assistance applications. Other examples include the Toshiba TMPV75 and TMPV76 image recognition processors and the NVIDIA DRIVE PX Xavier platform [1]. In addition, the NVIDIA Jetson Nano and Jetson TX2 platforms have been widely adopted for evaluating lane detection and object detection performance [2]. The NVIDIA Jetson AGX Xavier platform has also been integrated with millimeter-wave radar and RGB camera sensors for intelligent roadside units (RSUs) in traffic systems [3], as well as for in-vehicle driver monitoring and driver status detection applications [4,5].

Table 1 compares the proposed camera system with selected embedded vision platforms in terms of recognition performance, throughput, power, dimensions, and cost. The studies differ in application scenarios, AI models and algorithms, datasets, and evaluation metrics, limiting direct one-to-one comparison. Power values represent either reference profiles or measured consumption, while dimensions refer to IC packages, modules, or carrier boards. Prices are historical references under the stated purchasing conditions and do not represent current quotations or complete camera manufacturing costs [2,6,7,8,9,10,11,12,13,14,15,16,17,18,19,20,21].

Driven by considerations such as network bandwidth limitations, real-time response requirements, data latency, and personal privacy, inference and recognition tasks have gradually shifted from cloud servers toward local and edge devices. The objective is to perform image acquisition, recognition, decision-making, and command execution directly at the front-end device, while transmitting only event notifications and backup data to the central management system.

**Figure 1:**
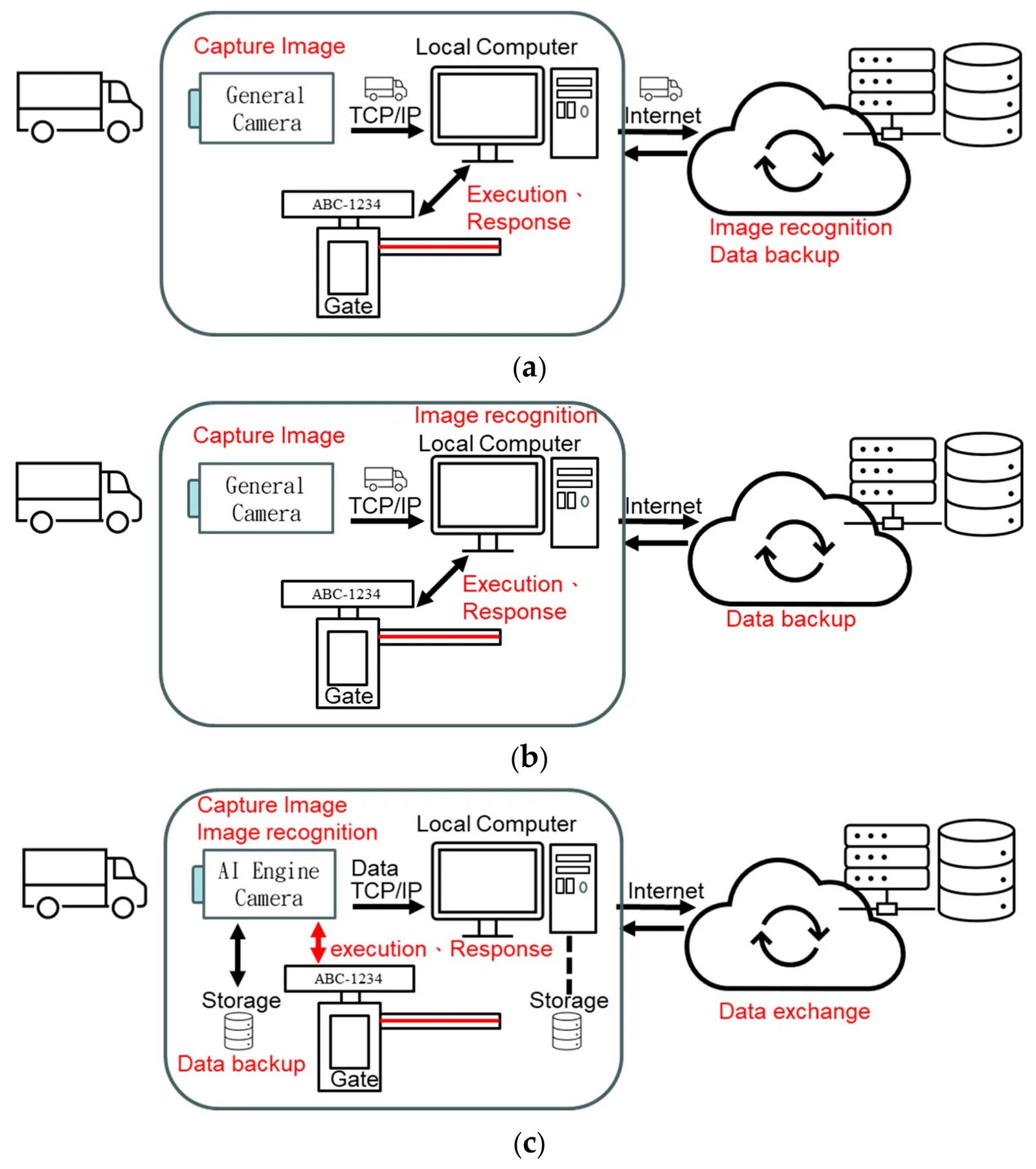
Comparison of image recognition architectures: (**a**) Cloud computing; (**b**) Local computing; (**c**) Edge computing.

Conventional road surveillance and parking management systems typically transmit camera images through multiple network layers to centralized servers for image recognition, control execution, and data storage, as illustrated in Figure 1a, or use a local server for image recognition instead, as shown in Figure 1b. In contrast, edge AI vision chips can perform recognition and decision-making locally at the device’s front end while simultaneously storing image data on local storage devices for backup and archival purposes. Relevant data can then be uploaded during periods of low network utilization. This approach significantly reduces network bandwidth consumption, transmission latency, and energy usage while enabling rapid low-latency real-time responses, as illustrated in Figure 1c.

Although embedded edge AI has been extensively investigated for real-time computer vision and intelligent transportation applications, existing studies have largely focused on recognition algorithms, inference performance, and hardware acceleration. Comparatively fewer studies have addressed the integrated system-level validation of a complete embedded AI camera, including AI inference, electrical characteristics, RF performance, electromagnetic compatibility, and practical field recognition. Therefore, this study focuses on the design, implementation, and experimental validation of a QCS610-based embedded edge AI camera.

This study utilizes Qualcomm’s QCS610 intelligent vision edge computing platform [22] to implement an embedded smart camera system. The QCS610 integrates a multicore, low-power processing architecture with an AI acceleration engine, an image signal processor (ISP), and communication interfaces. According to the platform specifications, it provides approximately 1 tera-operation per second (TOPS) of AI computing capability. These capabilities enable on-device image acquisition, recognition, decision-making, and control, while processed information is transmitted to a central management system.

Despite these advances, many existing studies have primarily focused on recognition algorithms and inference performance, whereas comprehensive system-level evaluations of complete embedded AI camera hardware—including high-speed signal integrity, RF transmitter performance, radiated emissions, and practical on-device AI operation—remain relatively limited. This study addresses this gap through the design, implementation, and experimental evaluation of a complete embedded edge AI camera system.

Specifically, the proposed system integrates circuit design, high-speed signal routing, and PCB stack-up design with experimental assessments of signal integrity, conducted RF transmitter performance, and radiated emissions. Vehicle type recognition and license plate recognition are implemented as an application demonstration to evaluate the practical operation of the developed camera system.

## 2. Related Work

This section reviews the literature relevant to embedded edge AI camera systems from three perspectives. First, recent developments in embedded edge AI vision and resource-constrained inference are reviewed. Second, edge AI applications for vehicle type detection and license-plate recognition are discussed. Third, representative embedded AI platforms and system-level validation issues are examined. This review provides the basis for identifying the research gap and positioning the contributions of the present QCS610-based embedded AI camera system.

### 2.1. Embedded Edge AI Vision Systems

Some studies have investigated embedded edge AI systems for real-time computer vision applications. By performing inference close to the image sensor, edge-based vision systems can reduce communication latency and dependence on centralized computing resources [23]. However, practical embedded AI deployment is constrained by computational capability, memory resources, power consumption, and inference latency. Consequently, lightweight neural-network architectures, hardware acceleration, and model-optimization techniques such as quantization have become important for resource-constrained edge devices [24].

In addition to recognition accuracy, embedded AI vision systems are commonly evaluated in terms of inference throughput, model complexity, memory footprint, and energy efficiency [25]. These factors are particularly important for compact camera systems, where computational performance must be balanced against power consumption and physical implementation constraints.

### 2.2. Edge AI for Vehicle Type and License-Plate Recognition

Edge AI has been increasingly applied to intelligent transportation systems, including vehicle detection, traffic monitoring, parking management, and automatic license-plate recognition (ALPR) [26]. Previous studies have demonstrated that vehicle and license-plate recognition can be performed directly on embedded computing platforms, reducing the need to transmit continuous image streams to centralized servers. Multi-stage recognition architectures have also been developed to integrate vehicle detection, license-plate localization, and optical character recognition within an edge inference pipeline [27].

Existing edge-based ALPR studies have evaluated recognition performance under practical traffic or parking environments, and some studies have further considered different illumination conditions, including daytime and nighttime scenarios [28]. These studies demonstrate the feasibility of embedded vehicle and license plate recognition, with a primary focus on recognition algorithms, inference speed, and recognition performance.

The present study focuses on the design, implementation, and experimental validation of an integrated embedded edge AI camera system, including hardware development, signal integrity assessment, RF transmitter performance evaluation, and radiated emissions testing. Vehicle type detection and license plate recognition serve as an application demonstration to assess the practical capabilities of the developed system.

### 2.3. Embedded AI Platforms and System-Level Validation

The selection of an embedded AI platform involves trade-offs among computational capability, inference throughput, power consumption, memory resources, physical size, and cost. Previous studies have therefore compared edge-computing devices using performance- and energy-related metrics, including inference throughput, power consumption, and cost efficiency [29]. Nevertheless, direct comparisons of recognition accuracy across different platforms should be interpreted cautiously because the reported results depend on the neural-network architecture, dataset, input resolution, preprocessing procedure, numerical precision, inference framework, and evaluation protocol.

Beyond AI inference performance, the implementation of a complete embedded AI camera also requires reliable high-speed digital interfaces, wireless communication, and power management. High-speed camera and display interfaces, such as MIPI D-PHY, require careful consideration of high-speed differential transmission, noise, jitter, and electromagnetic interference [30]. More generally, impedance discontinuities, conductor and dielectric losses, vias, connectors, and other interconnect structures can cause signal attenuation and reflection in high-speed PCB interconnections. Frequency-domain analyses based on scattering parameters can therefore be employed to evaluate the transmission characteristics and signal integrity of these interconnections [31]. Furthermore, signal-integrity design is closely related to electromagnetic compatibility because high-speed PCB interconnections can also contribute to radiated emissions [32].

Although previous studies have demonstrated substantial progress in embedded AI inference, intelligent transportation vision, edge-computing platforms, and high-speed hardware design, comparatively fewer studies have presented an integrated system-level evaluation of a complete embedded AI camera that combines AI inference with high-speed signal-integrity verification, wireless RF characterization, radiated-emission testing, power-consumption evaluation, and real-world recognition experiments. The present study addresses this gap through the design, implementation, and experimental validation of a QCS610-based embedded edge AI camera.

## 3. System Architecture and Hardware Design

**Figure 2:**
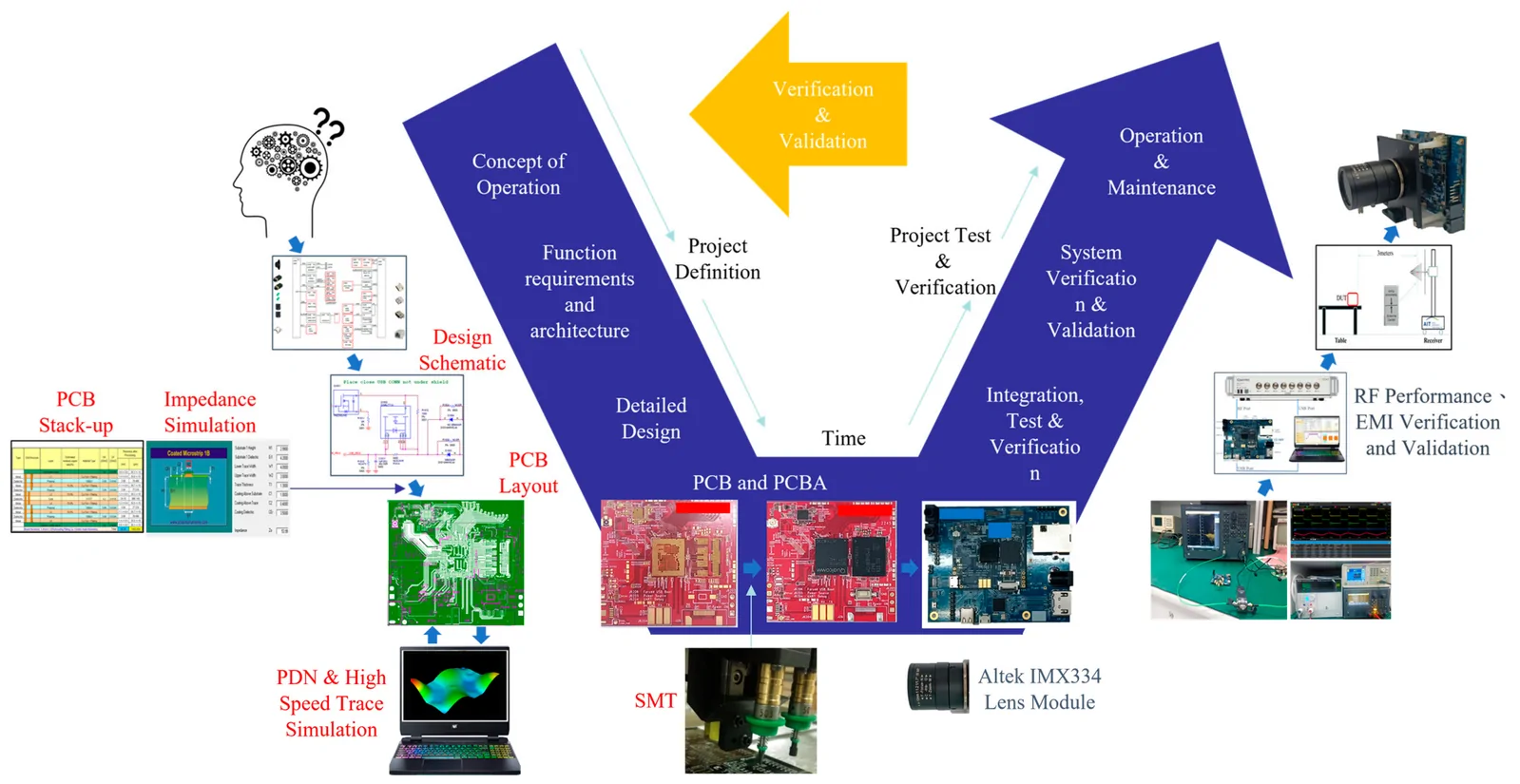
Product design process. The arrows indicate the sequence of the design process.

Figure 2 illustrates the proposed V-model-based development process for the embedded edge AI camera system [33,34,35]. The process includes circuit and schematic design, printed circuit board (PCB) layout and simulation, hardware implementation and testing, radio frequency (RF) performance verification, spectrum analysis, and radiated emission pre-compliance evaluation.

### 3.1. System Overview and Functional Definition

**Figure 3:**
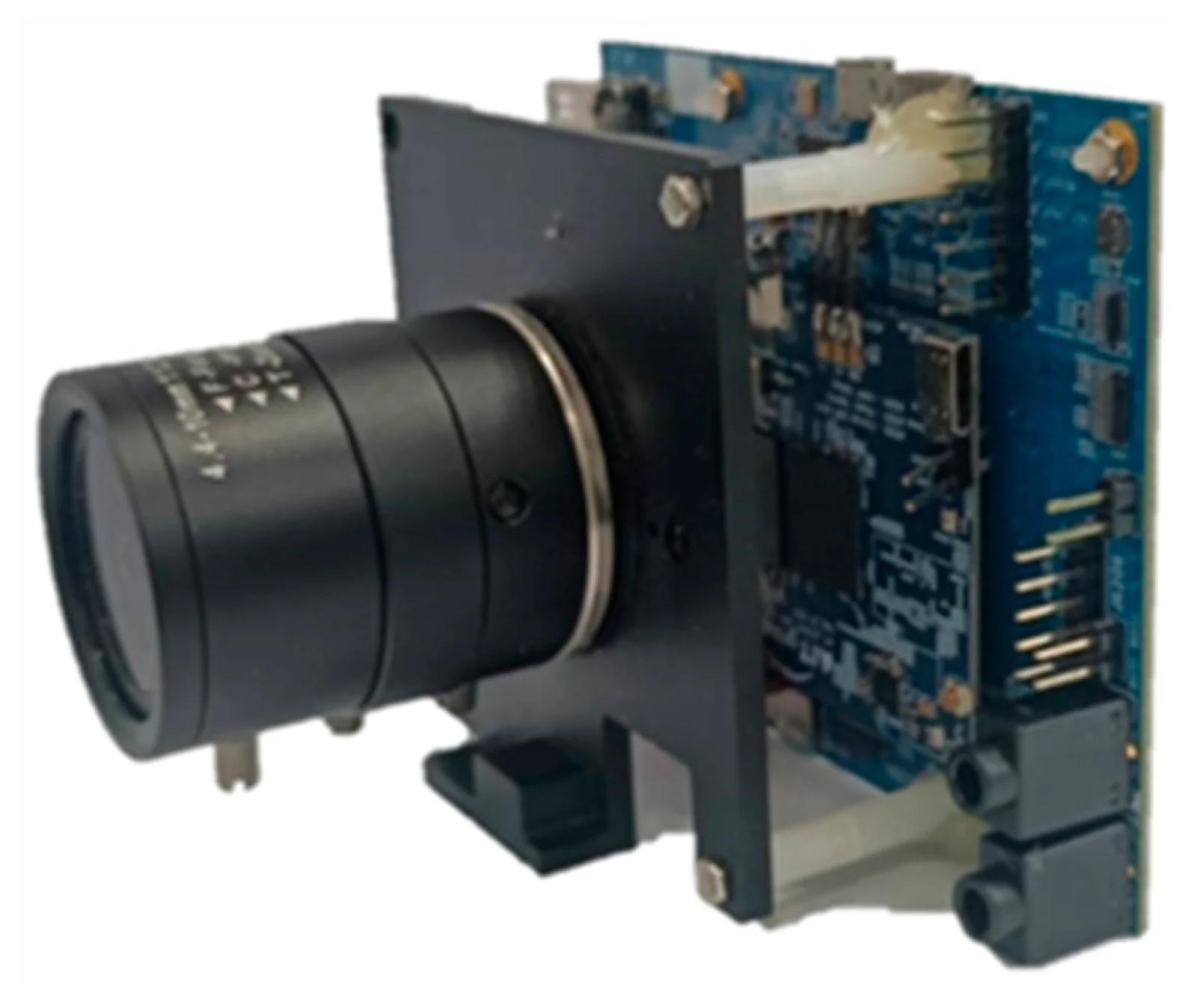
Proposed system.

The proposed embedded edge AI system is implemented on a Digital Signal Processor (DSP)-based multi-core edge computing platform and supports image processing at a resolution of 1280 × 960 pixels. The system performs image acquisition, intelligent recognition, local decision-making, and event reporting directly on the device, thereby reducing network bandwidth requirements and response latency. Figure 3 illustrates the proposed system.

### 3.2. Circuit Design and Schematic Development

**Figure 4:**
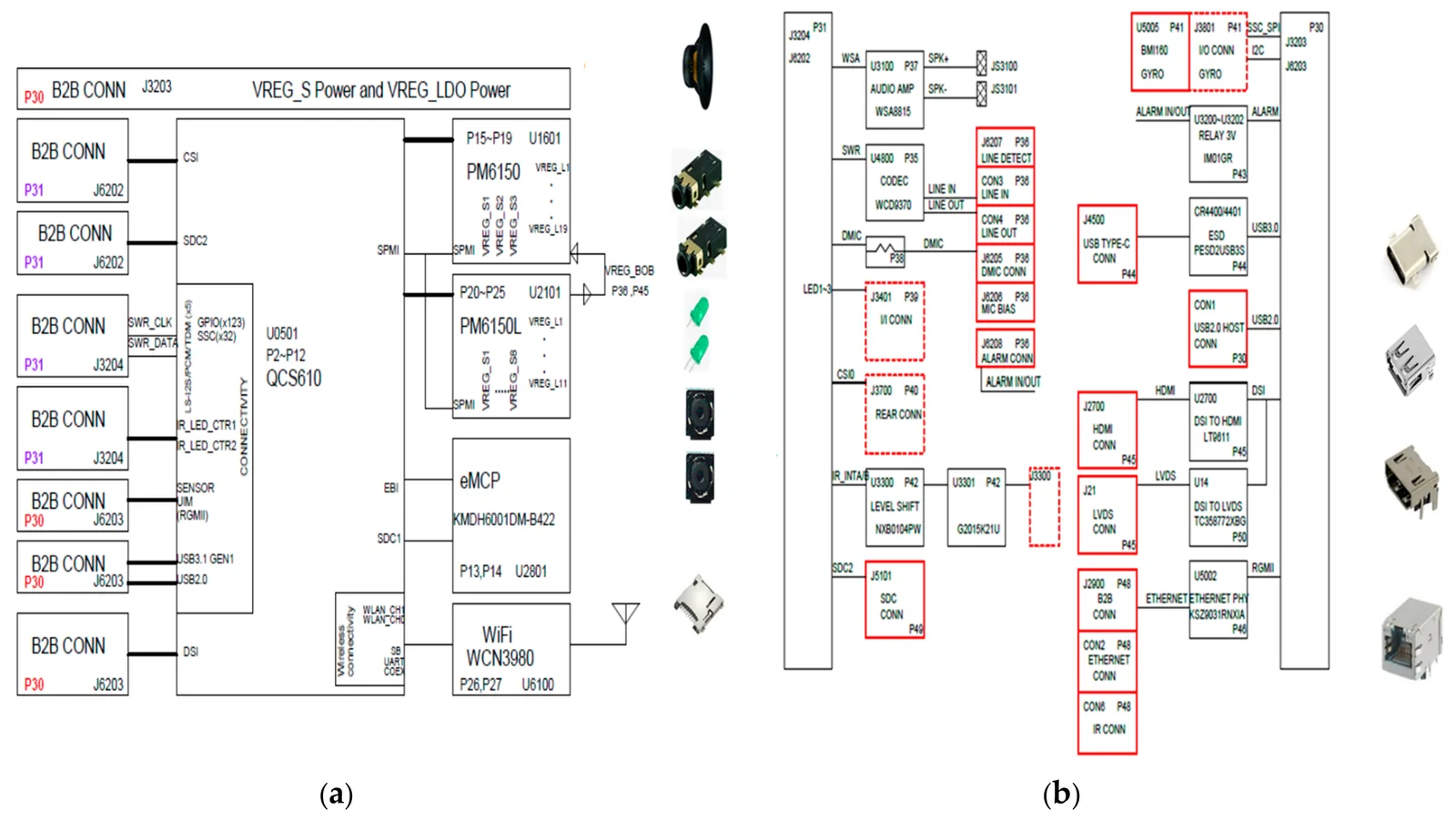
System-level schematic of the QCS610-based embedded edge AI camera system: (**a**) core processing and power management architecture; (**b**) peripheral interface and external connector architecture.

During the circuit design stage, Cadence OrCAD Capture CIS [36] was employed to develop the system schematic based on the functional requirements, interface specifications, logic-level definitions, and power distribution architecture of the proposed embedded edge AI camera system. The system-level schematic was organized into functional blocks to clearly represent the interconnections among the major hardware modules, as shown in Figure 4 which illustrates the overall hardware architecture of the QCS610-based embedded edge AI camera system. In Figure 4a, the QCS610 SoC is located at the center of the system architecture and serves as the main processing unit. On the left side, multiple board-to-board (B2B) connectors provide interfaces for external modules through CSI, DSI, USB, SDC, SoundWire, sensor interrupt, and control signals. On the right side, the QCS610 interfaces with the PM6150 and PM6150L power management integrated circuits (PMICs), the eMCP memory device, and the WCN3960 Wi-Fi module. The PMICs generate the regulated VREG_S and VREG_L power rails required by the SoC and associated components and are controlled through the System Power Management Interface (SPMI). The eMCP provides the memory and storage resources, while the WCN3960 provides wireless connectivity through the external antenna.

Figure 4b provides a more detailed representation of the peripheral interfaces connected through the B2B connections shown in Figure 4a. On the left side, the system interfaces with several external peripherals, including the audio amplifier, audio codec, microphone, rear-camera interface, SD card interface, USB Type-C connector, and display-related connectors. On the right side, additional interfaces are provided for display conversion, Ethernet communication, USB host connectivity, and general-purpose I/O. In particular, DSI signals from the main processing system can be converted to HDMI or LVDS through dedicated interface-conversion circuits, allowing the system to support different display interfaces. The Ethernet PHY provides the physical-layer interface for wired network communication, while the remaining connectors provide access to USB and other external I/O functions.

Together, Figure 4a,b illustrate the hierarchical organization of the hardware system. Figure 4a presents the core processing, power management, memory, wireless communication, and primary B2B interfaces, whereas Figure 4b expands these B2B interfaces to show how the main system is connected to the external peripheral circuits and physical connectors.

Upon completion of the schematic design, a Design Rule Check (DRC) was performed to verify design integrity and identify potential errors or rule violations. Once the identified issues had been resolved and the design had passed the required checks, the verified design data were exported for the subsequent PCB layout stage.

### 3.3. PCB Layout and Signal Integrity Design

During the high-speed PCB design stage, signal integrity (SI) is a critical consideration for ensuring reliable signal transmission in embedded edge AI systems. As operating frequencies increase, PCB traces can no longer be regarded as ideal conductors, and transmission-line effects become significant. Impedance discontinuities, signal reflections, insertion loss, and electromagnetic coupling can degrade signal integrity and overall system performance. At high frequencies, the skin and proximity effects alter the current distribution within conductors. Impedance-controlled routing, analytical modeling, and numerical simulation are essential to preserve signal integrity and reduce unintended electromagnetic emissions in high-speed embedded hardware systems.

**Figure 5:**
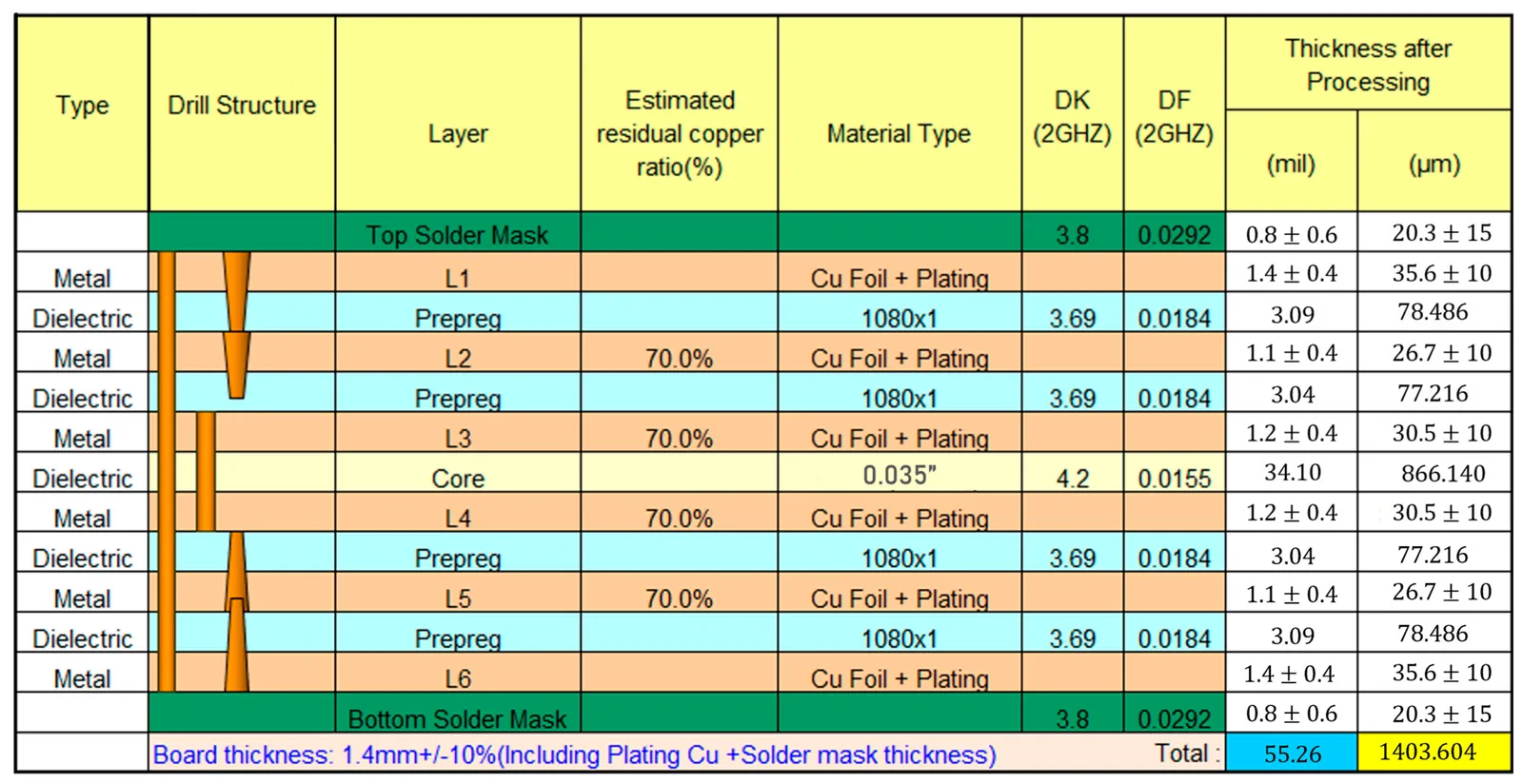
PCB stack-up. The orange, blue, and green indicate metal layers, dielectric layers, and solder mask layers, respectively.

Based on the system architecture, component placement, PCB stack-up planning, and routing design were performed using PADS version 9.3 [37]. Figure 5 is the PCB Stack-up. Table 2 summarizes the PCB stack-up configuration and interface requirements. To ensure manufacturability and compliance with the design specifications, the stack-up structure and fabrication constraints were reviewed jointly with the PCB manufacturer. The impedance characteristics of critical high-speed interfaces were evaluated using the Polar Si9000 simulation tool version 7.1, while their signal transmission performance was assessed through S-parameter analysis and experimental measurements.

#### 3.3.1. Skin and Proximity Effects

In high-frequency PCB design, signal transmission characteristics are influenced by the skin and proximity effects [38,39,40]. As the operating frequency increases, current tends to concentrate near the conductor surface because of the skin effect, reducing the effective conducting area and increasing AC resistance and conductor loss. In addition, the proximity effect redistributes current according to the electromagnetic fields generated by adjacent conductors and reference planes [41,42,43,44,45]. These effects can increase transmission loss and degrade signal integrity in high-speed interconnections [46,47,48,49].

In the proposed embedded edge AI camera, these high-frequency effects were considered in the PCB implementation through the selection of the PCB stack-up, controlled trace geometry, and continuous ground-reference planes for the high-speed signal paths. The high-speed traces were designed with controlled impedance, and the resulting transmission characteristics were subsequently evaluated through signal-integrity simulation and experimental measurement. Thus, the theoretical considerations in this section were used to guide the practical PCB implementation rather than being treated solely as general theoretical background.

#### 3.3.2. Transmission Line Theory and Impedance Matching

According to transmission-line theory [47], the characteristic impedance of a transmission line is determined by its distributed resistance (*R*), inductance (*L*), conductance (*G*), and capacitance (*C*), and angular frequency (*ω*). The characteristic impedance (*Z*_0_) can be expressed as

(1) $$Z_{0}=\sqrt{\frac{R+j\omega L}{G+j\omega C}}$$

For practical high-speed PCB interconnects operating under low-loss conditions (*R* ≪ *ωL*; *G* ≪ *ωC*), the characteristic impedance can be approximated as

(2) $$Z_{0}\approx \sqrt{\frac{L}{C}}$$

Ideally, the source impedance (*Z_s_*), characteristic impedance (*Z*_0_), and load impedance (*Z_L_*) satisfy

*Z_s_* = *Z*_0_ = *Z_L_* (3)

The magnitude of the reflected wave is quantified by the reflection coefficient (*Γ*), which is defined as

(4) $$\Gamma =\frac{Z_{L}-Z_{0}}{Z_{L}+Z_{0}}$$

When *Z_L_* = *Z*_0_, *Γ* = 0, the reflection coefficient becomes zero, indicating that no signal reflection occurs. The severity of standing waves caused by impedance mismatch can be evaluated using the Voltage Standing Wave Ratio (*VSWR*),

(5) $$\mathrm{VSWR}=\frac{1+|\Gamma |}{1-|\Gamma |}$$

where a lower *VSWR* value indicates better impedance matching and improved signal transmission performance.

#### 3.3.3. Impedance and Signal Integrity Simulation

To ensure reliable high-speed signal transmission, transmission-line effects and impedance control must be considered during the PCB design stage. Based on transmission-line theory and IPC design guidelines [50,51,52,53,54,55], the characteristic impedance of PCB traces can be estimated using analytical expressions for different routing structures.

For a single-ended microstrip line, the characteristic impedance can be approximated by

(6) $$Z_{o}=\frac{87}{\sqrt{\varepsilon_{r}+1.41}}\times \ln\left(\;\frac{5.98h}{0.8w+t}\right)$$

For a strip-line structure, the characteristic impedance is given by

(7) $$Z_{o}\approx \frac{60}{\sqrt{\varepsilon_{r}}}\times \ln\left(\;\frac{1.9(2h+t)}{0.8w+t}\right)$$

For edge-coupled differential microstrip traces, the differential impedance can be estimated using

(8) $$Z_{d}\approx \frac{174}{\sqrt{\varepsilon_{r}+1.41}}\times \ln\left(\;\frac{5.98h}{\left(0.8w+t\right)})\times (1-0.48e^{\left(-0.96\frac{s}{h}\right)}\right)$$

Similarly, for edge-coupled strip-line structures, the differential impedance can be approximated by

*Z_d_* ≈ 2*Z_o_*[1 − 0.347*e*^−2.9*s*/(2*h*+*t*)^] (9)

where *Z_o_* is the single-ended characteristic impedance, *Z_d_* is the differential impedance, *ε_r_* is the relative permittivity of the PCB substrate, *h* is the dielectric thickness, *w* is the trace width, *t* is the copper thickness, and *s* is the spacing between differential traces.

Based on the stack-up configuration and routing constraints summarized in Table 2, the characteristic impedances of the critical high-speed interfaces were estimated using the analytical expressions described above. The resulting trace dimensions and routing parameters were subsequently verified using the Polar Si9000 transmission-line simulation tool.

**Figure 6:**
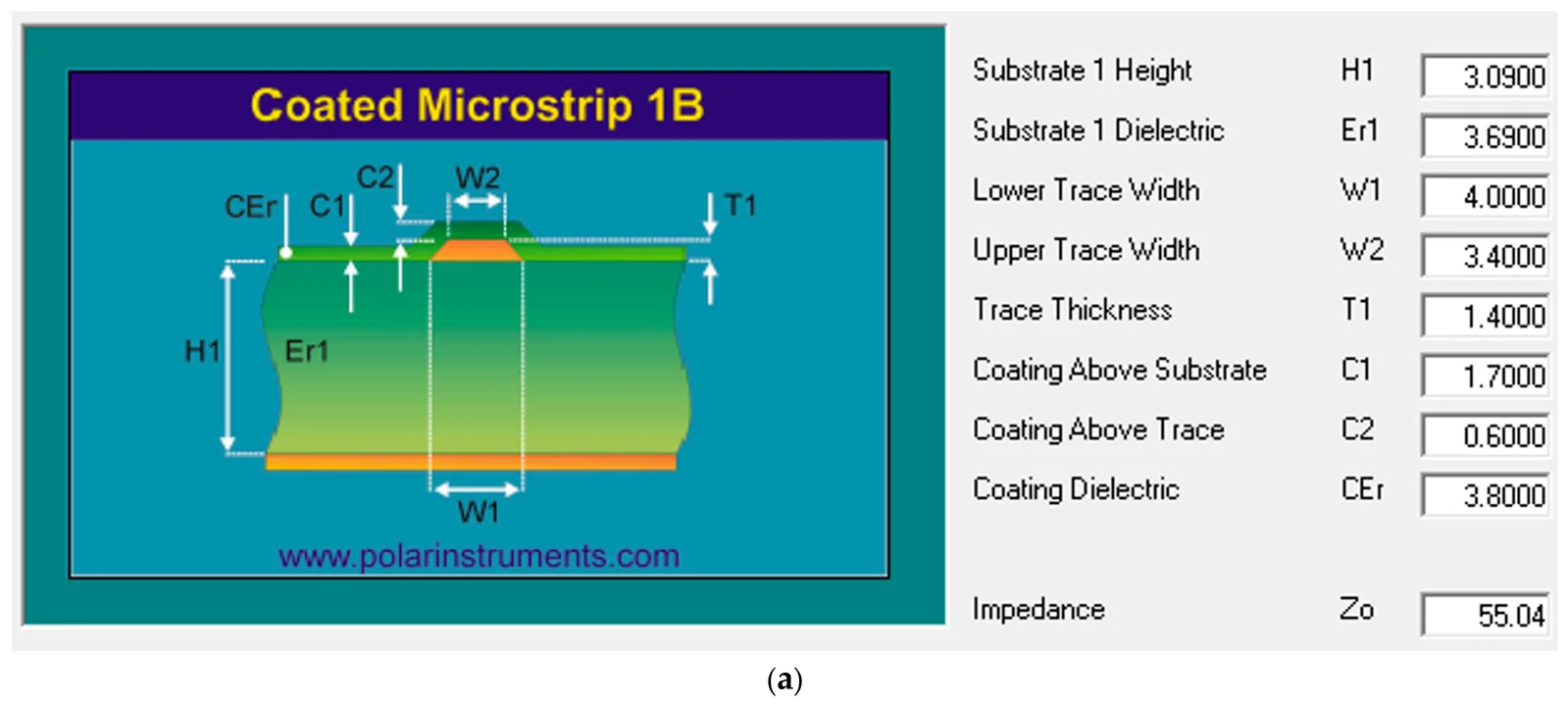

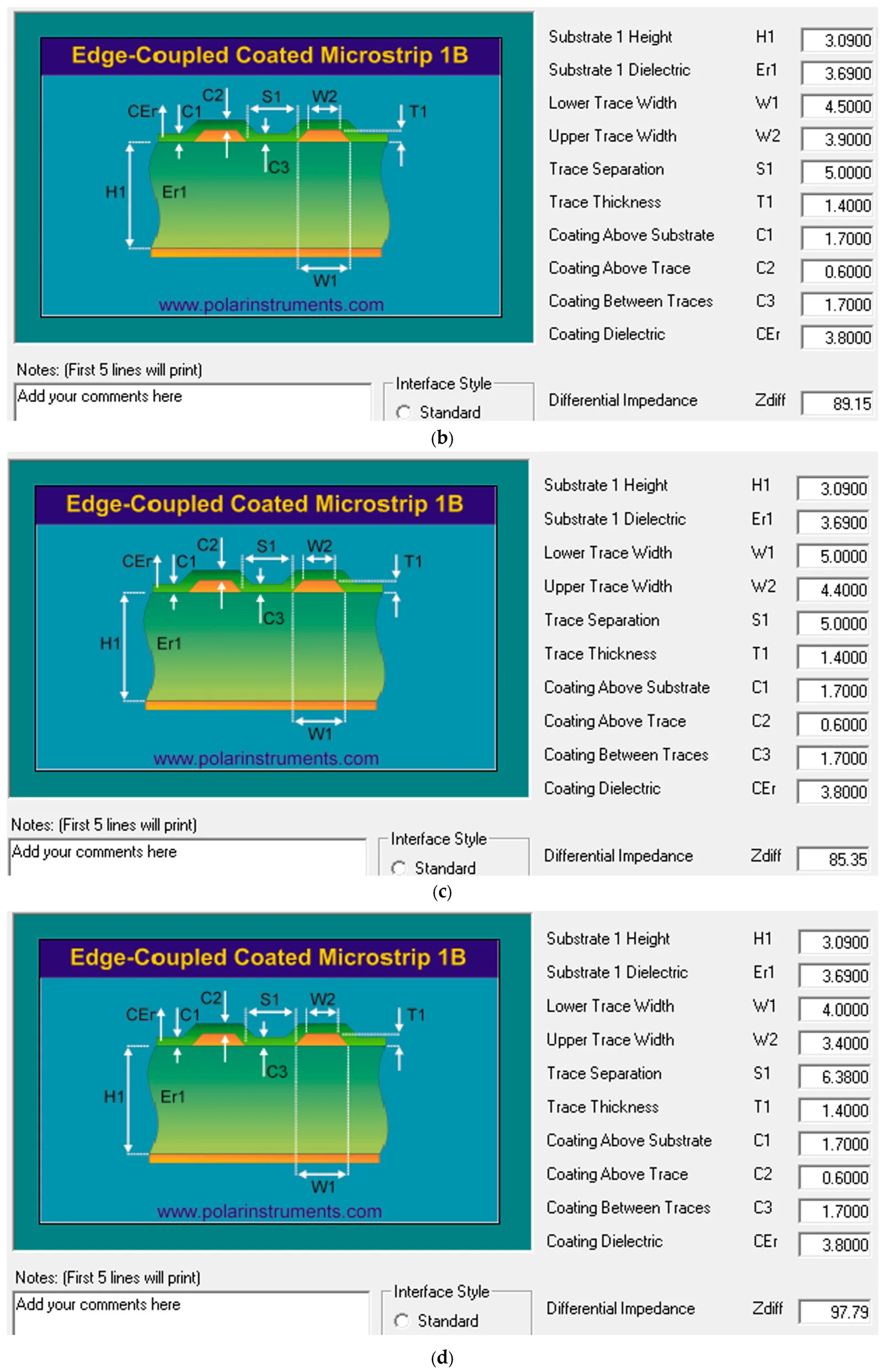
Impedance simulations of the outer-layer PCB traces: (**a**) Coated microstrip for single-ended signals, with W_1_ = 4.0 mil, W_2_ = 3.4 mil; (**b**) Edge-coupled coated microstrip for USB differential signals, with W_1_ = 4.5 mil, W_2_ = 3.9 mil, S_1_ = 5.0 mil; (**c**) Edge-coupled coated microstrip for MIPI differential signals, with W_1_ = 5.0 mil, W_2_ = 4.4 mil, S_1_ = 5.0 mil; (**d**) Edge-coupled coated microstrip for Ethernet/HDMI differential signals, with W_1_ = 4.0 mil, W_2_ = 3.4 mil, S_1_ = 6.38 mil.

Figure 6 shows the impedance simulation results for the outer-layer PCB traces, where W_1_ and W_2_ denote the lower and upper trace widths, respectively, and S_1_ denotes the edge-to-edge spacing between the differential traces. Figure 6a is a coated microstrip for single-ended signals with W_1_ = 4.0 mil, W_2_ = 3.4 mil; Figure 6b is an edge-coupled coated microstrip for USB differential signals with W_1_ = 4.5 mil, W_2_ = 3.9 mil, S_1_ = 5.0 mil; Figure 6c is for MIPI with W_1_ = 5.0 mil, W_2_ = 4.4 mil, S_1_ = 5.0 mil; and Figure 6d is for Ethernet/HDMI with W_1_ = 4.0 mil, W_2_ = 3.4 mil, and S_1_ = 6.38 mil.

**Figure 7:**
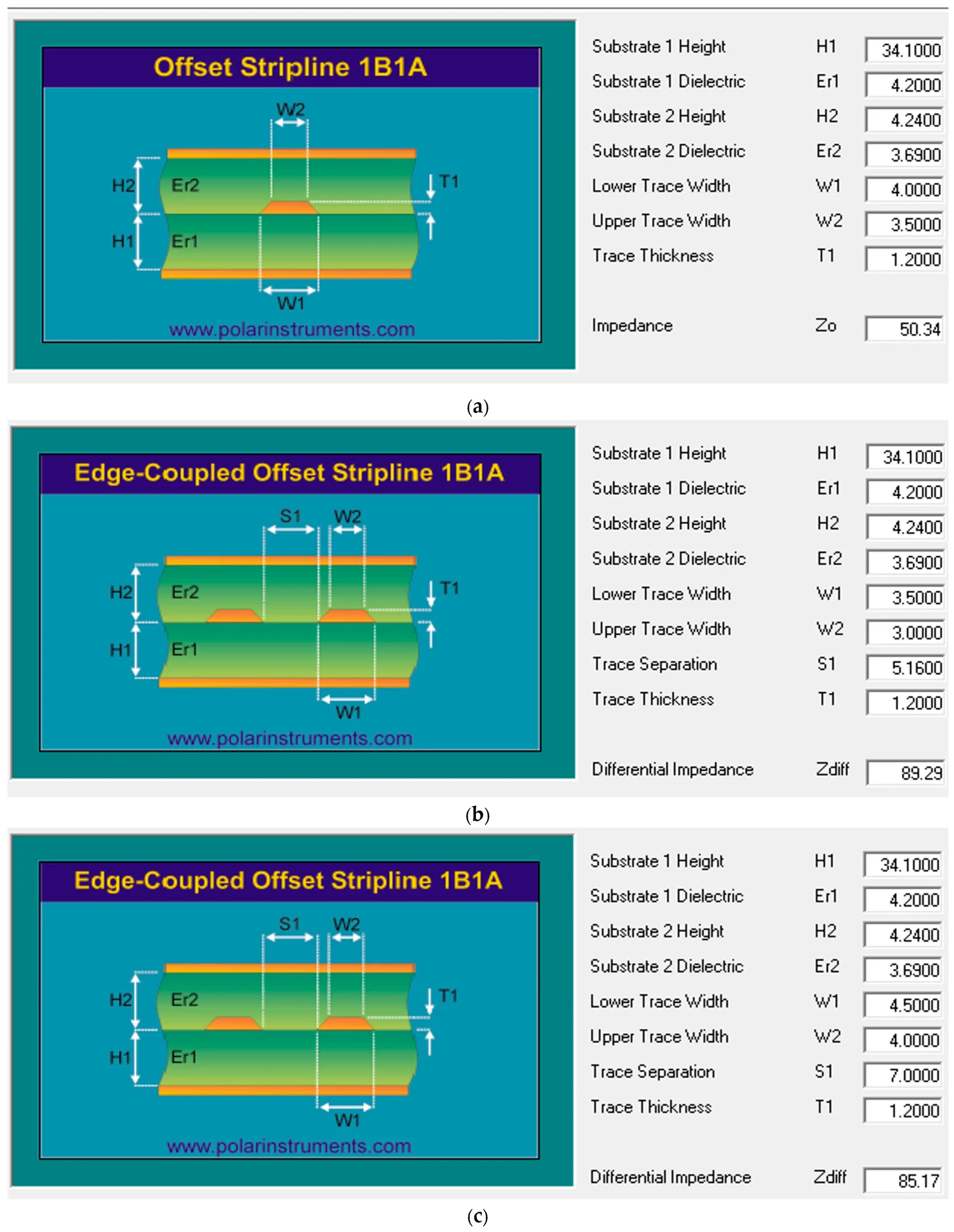

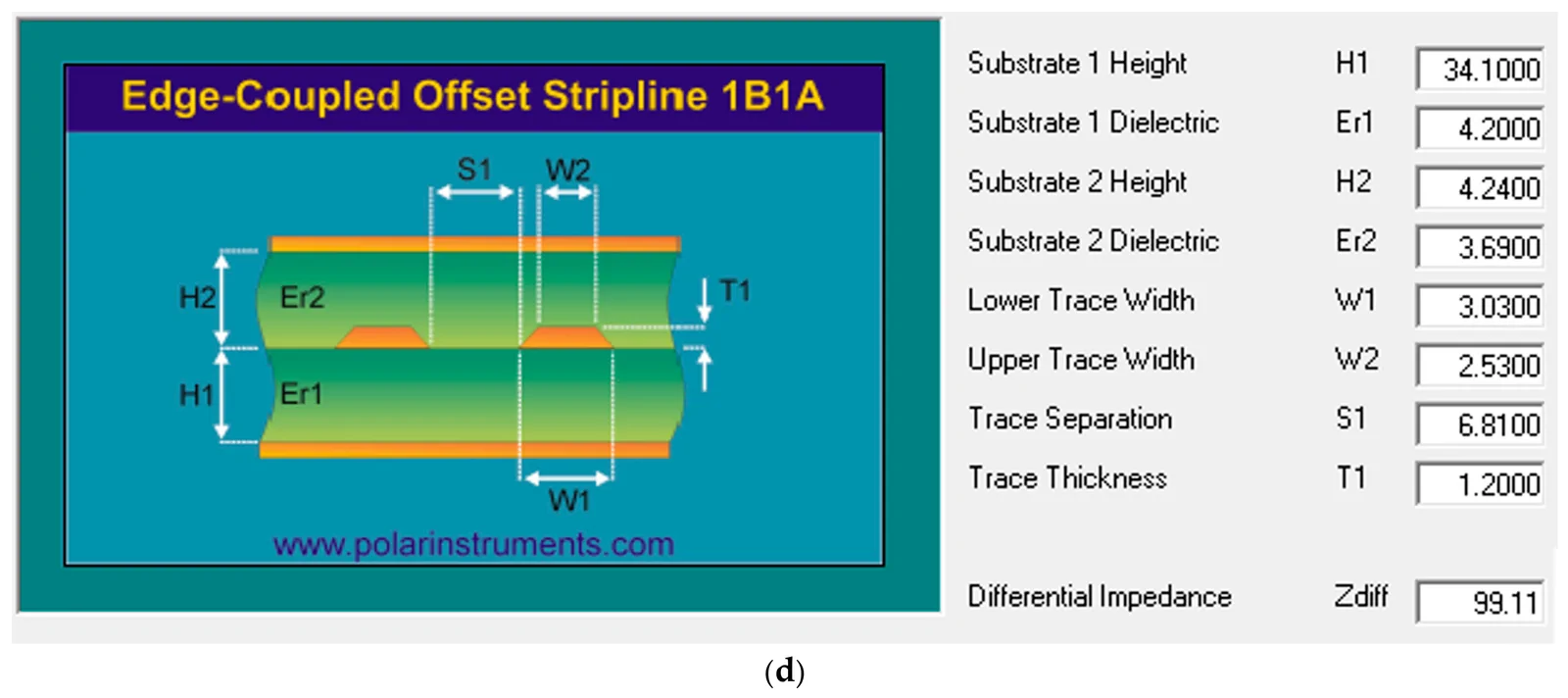
Impedance simulations of the inner-layer PCB traces: (**a**) Offset stripline for single-ended signals with W_1_ = 4.0 mil, W_2_ = 3.5 mil; (**b**) Edge-coupled offset stripline for USB differential signals with W_1_ = 3.5 mil, W_2_ = 3.0 mil, S_1_ = 5.16 mil; (**c**) Edge-coupled offset stripline for MIPI differential signals with W_1_ = 4.5 mil, W_2_ = 4.0mil, S_1_ = 7.0 mil; (**d**) Edge-coupled offset stripline for Ethernet/HDMI differential signals with W_1_ = 3.03 mil, W_2_ = 2.53 mil, S_1_ = 6.81 mil.

Figure 7 is the impedance simulation results for the inner-layer PCB traces. Figure 7a is an offset stripline for single-ended signals with W_1_ = 4.0 mil, W_2_ = 3.5 mil; Figure 7b is an edge-coupled offset stripline for USB differential signals with W_1_ = 3.5 mil, W_2_ = 3.0 mil, S_1_ = 5.16 mil; Figure 7c is for MIPI with W_1_ = 4.5 mil, W_2_ = 4.0 mil, S_1_ = 7.0 mil; and Figure 7d is for Ethernet/HDMI with W_1_ = 3.03 mil, W_2_ = 2.53 mil, S_1_ = 6.81 mil.

Table 3 summarizes the simulated impedance results and compares them with the target specifications for the high-speed interfaces. All evaluated transmission-line configurations satisfy the specified impedance tolerances, confirming the suitability of the selected routing parameters and PCB stack-up for the final hardware implementation.

Although these analytical models provide useful preliminary estimates of transmission-line impedance, their accuracy is limited by simplifying assumptions regarding conductor geometry and electromagnetic field distribution. Therefore, modern high-speed electronic systems increasingly rely on simulation-driven design methodologies to evaluate signal integrity (SI) and power integrity (PI) performance before hardware fabrication. Such simulations help identify potential design issues early in the development process, thereby reducing design iterations, shortening development cycles, and lowering overall development costs.

**Figure 8:**
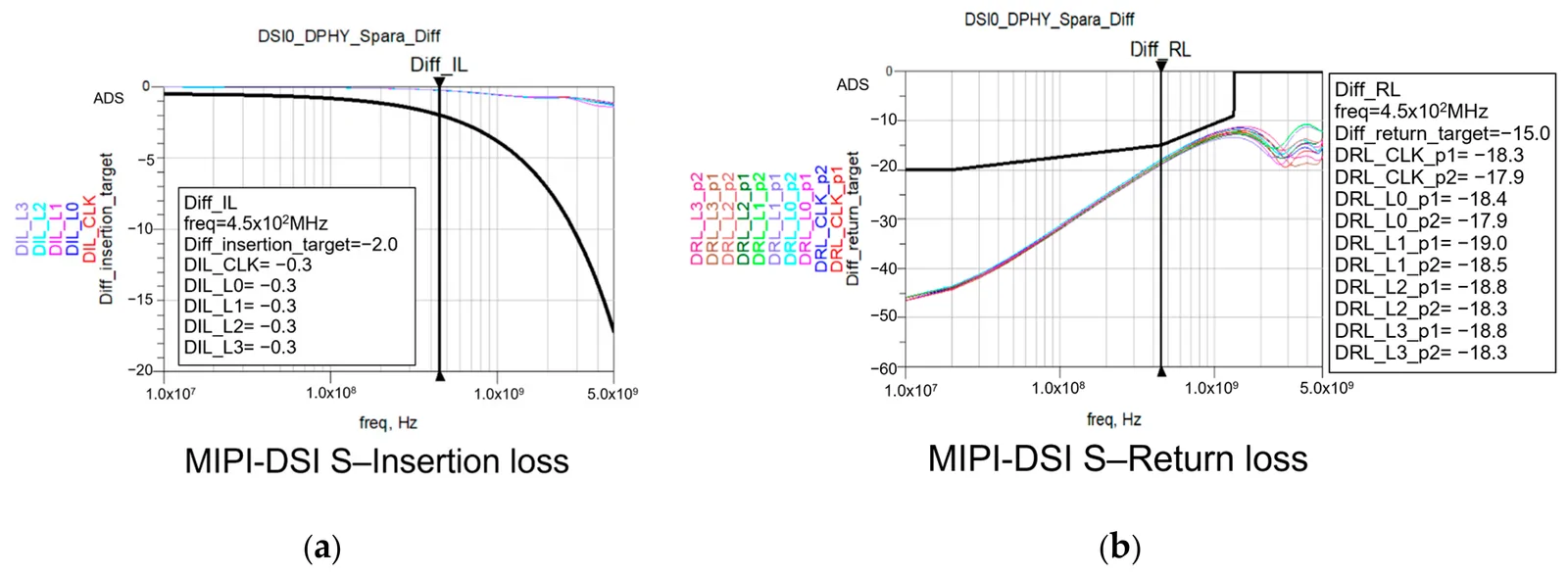
MIPI-DSI differential S-parameter simulation results: (**a**) Insertion loss; (**b**) Return loss.

Following the impedance verification, frequency-domain signal integrity simulations were performed for the MIPI Display Serial Interface (MIPI-DSI). Differential S-parameter analysis was conducted to evaluate the transmission and reflection characteristics of this high-speed interface. Figure 8 presents the simulated differential S-parameter characteristics of the MIPI-DSI interface: Figure 8a shows the differential insertion loss, whereas Figure 8b shows the differential return loss. The simulated responses satisfy their respective target limits over the evaluated frequency range, indicating that the MIPI-DSI interface meets the specified signal integrity requirements.

## 4. Experimental Setup and Evaluation Methods

**Figure 9:**
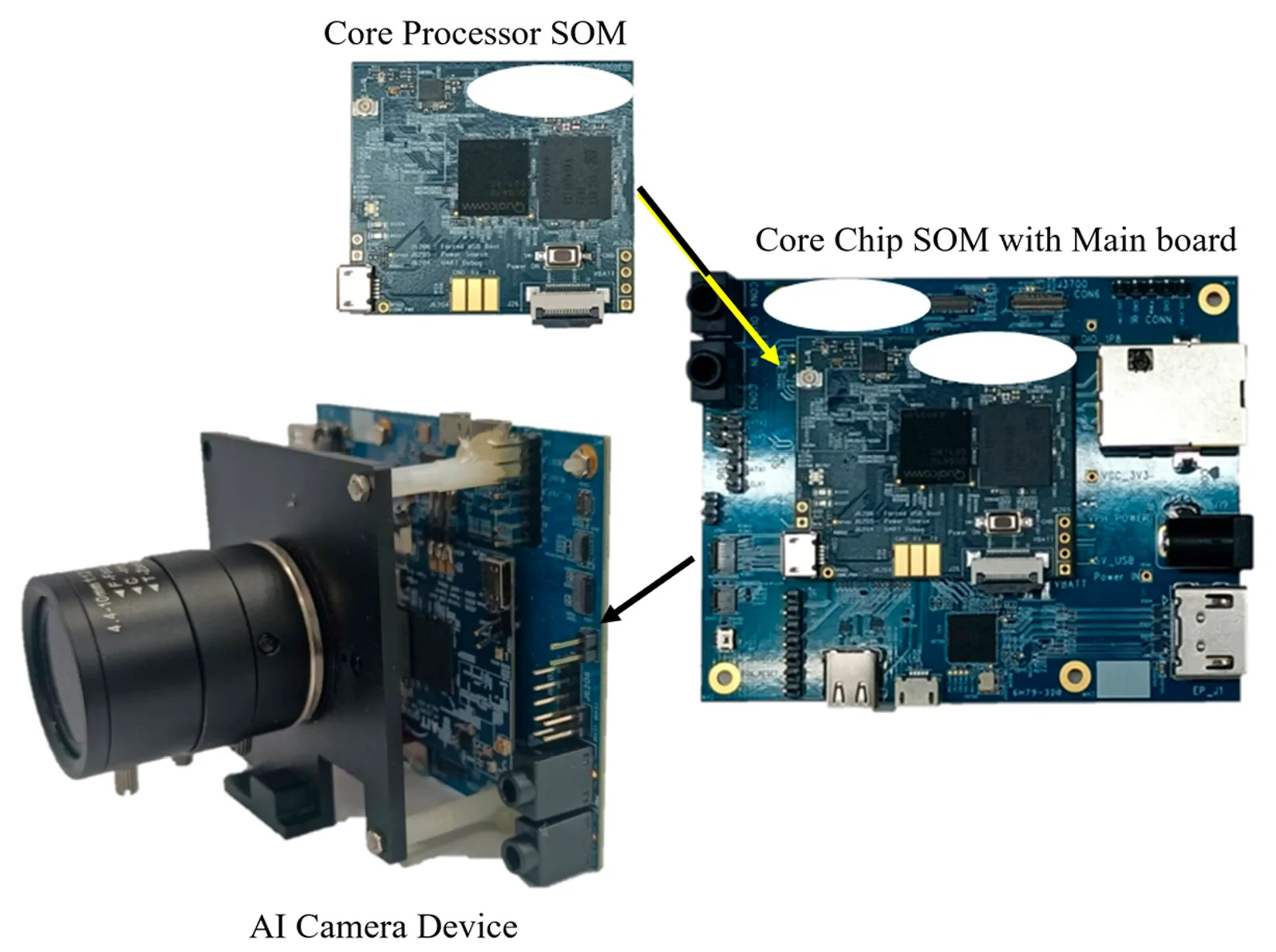
Hardware prototype of the proposed embedded edge AI camera system. The arrows indicate the location of the core processor SOM on the main board and its integration into the complete AI camera device.

To evaluate the performance of the proposed embedded edge AI camera system, a comprehensive experimental framework was established, including hardware verification, signal integrity measurements, RF performance evaluation, electromagnetic emission assessment, and intelligent recognition performance testing. Figure 9 illustrates the overall architecture of the developed platform. The proposed smart camera system integrates a camera module, a Qualcomm-based edge computing processor with a built-in artificial intelligence engine, memory devices, wireless communication modules, and associated high-speed interface circuits. The embedded platform performs on-device image acquisition, data processing, intelligent inference, and event reporting without relying on cloud-based computation.

A digital signal processor (DSP) integrated into the Qualcomm QCS610 SoC (Qualcomm Technologies, Inc., San Diego, CA, USA) was employed to execute image-processing and recognition tasks locally on the embedded platform. During the experiments, the image resolution was configured at 1280 × 960 pixels. Multiple intelligent transportation applications, including vehicle type detection and license plate recognition, were implemented to evaluate the practical performance of the proposed system.

The experimental procedures included signal integrity verification using a vector network analyzer (VNA; E5063A, Keysight Technologies, Santa Rosa, CA, USA), wireless communication performance evaluation, spectrum analysis, radiated emission measurements, and intelligent recognition performance assessment. The corresponding measurement setups and test methodologies are described in the following sections.

### 4.1. Signal Integrity Measurement Setup

**Figure 10:**
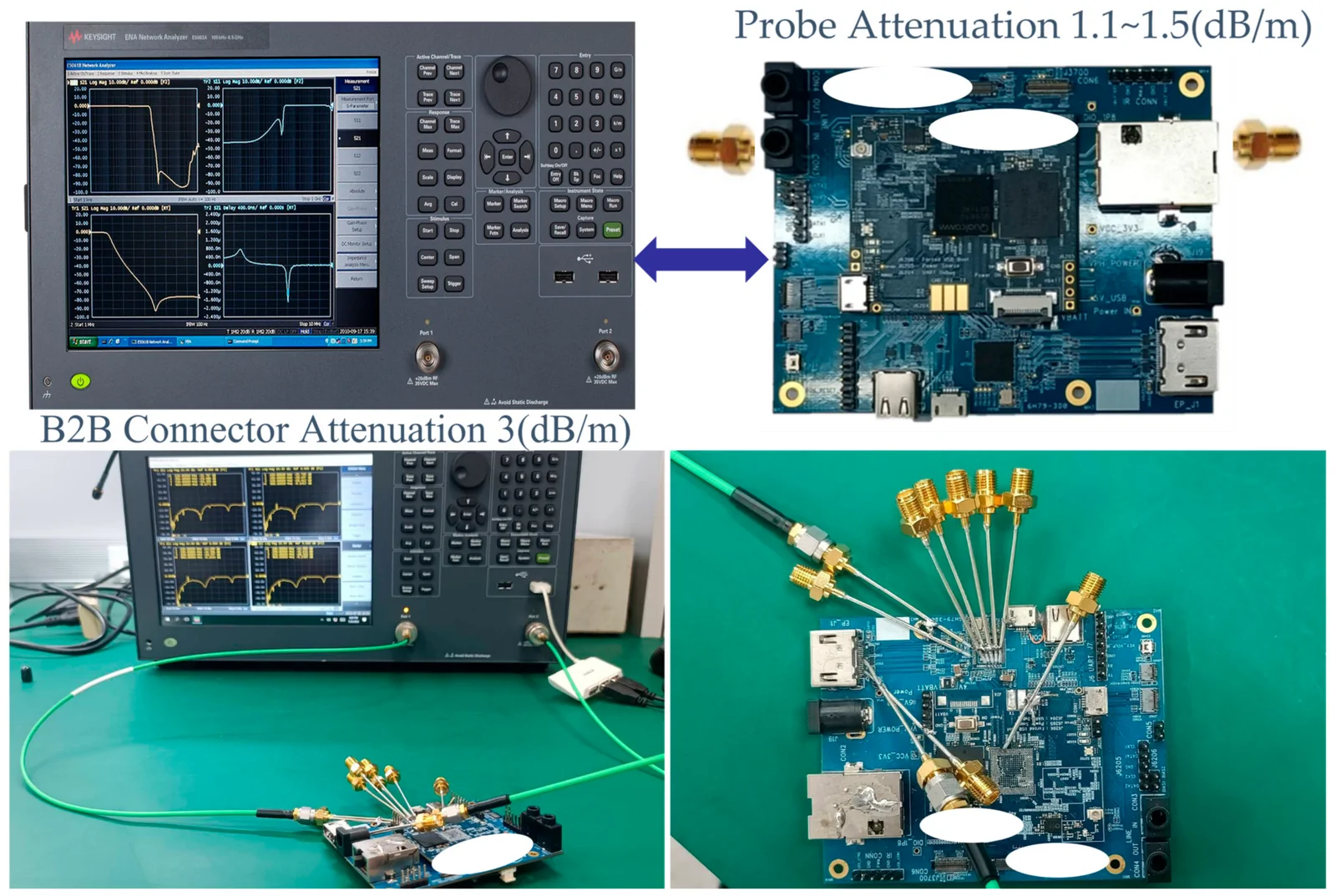
S-parameter measurement setup using a Vector Network Analyzer.

The signal integrity performance of the fabricated high-speed PCB was evaluated using a VNA and a high-bandwidth oscilloscope (XR104A, Keysight Technologies, Santa Rosa, CA, USA). The VNA was used to measure the S-parameters of critical high-speed interfaces, including the reflection coefficient (S_11_) and transmission coefficient (S_21_), in order to characterize impedance matching, return loss, insertion loss, and signal transmission performance. Figure 10 illustrates the measurement configuration.

To validate the PCB design methodology, the measured S-parameter results were compared with the corresponding simulation results. The correlation between simulation and measurement was used to verify the effectiveness of the impedance-controlled routing strategy and the accuracy of the signal integrity modeling process.

In addition, a digital oscilloscope was utilized to evaluate waveform quality under practical operating conditions. Key signal quality indicators, including signal amplitude, rise time, fall time, overshoot, undershoot, ringing, and timing stability, were examined to ensure reliable operation of the high-speed interfaces.

### 4.2. RF Verification Setup Using IQxel Multi-Wireless

**Figure 11:**
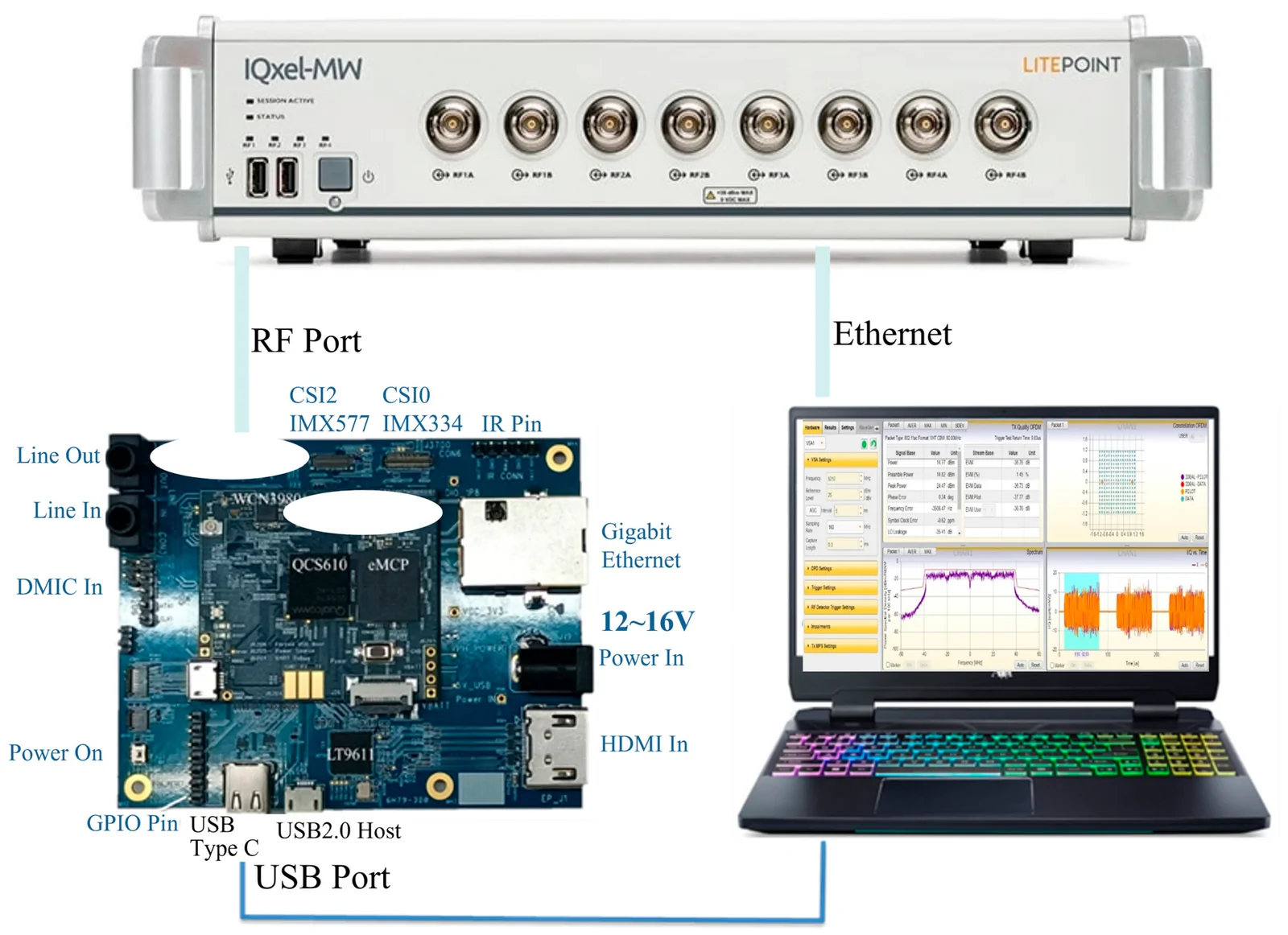
IQxel multi-wireless RF verification test setup.

The proposed embedded edge AI camera system supports both Wi-Fi and Bluetooth connectivity. To characterize the conducted RF transmitter performance of these wireless interfaces, the RF port of the developed prototype was connected to a LitePoint IQxel-MW (LitePoint Corporation, San Jose, CA, USA) wireless connectivity test system [56], as illustrated in Figure 11. The same LitePoint IQxel-MW test platform was used to verify both the Wi-Fi and Bluetooth transmitters integrated into the developed AI camera system.

For Wi-Fi, measurements were conducted in the 2.4 and 5 GHz bands to evaluate output power, spectral characteristics, and modulation quality. For Bluetooth, the measurements included transmit power, adjacent-channel power, GFSK eye-diagram analysis in Classic Basic Rate mode, and differential error vector magnitude (DEVM) analysis in Enhanced Data Rate mode. The measured results were subsequently compared with the corresponding specification limits.

### 4.3. Spectrum Analysis Measurement Setup

**Figure 12:**
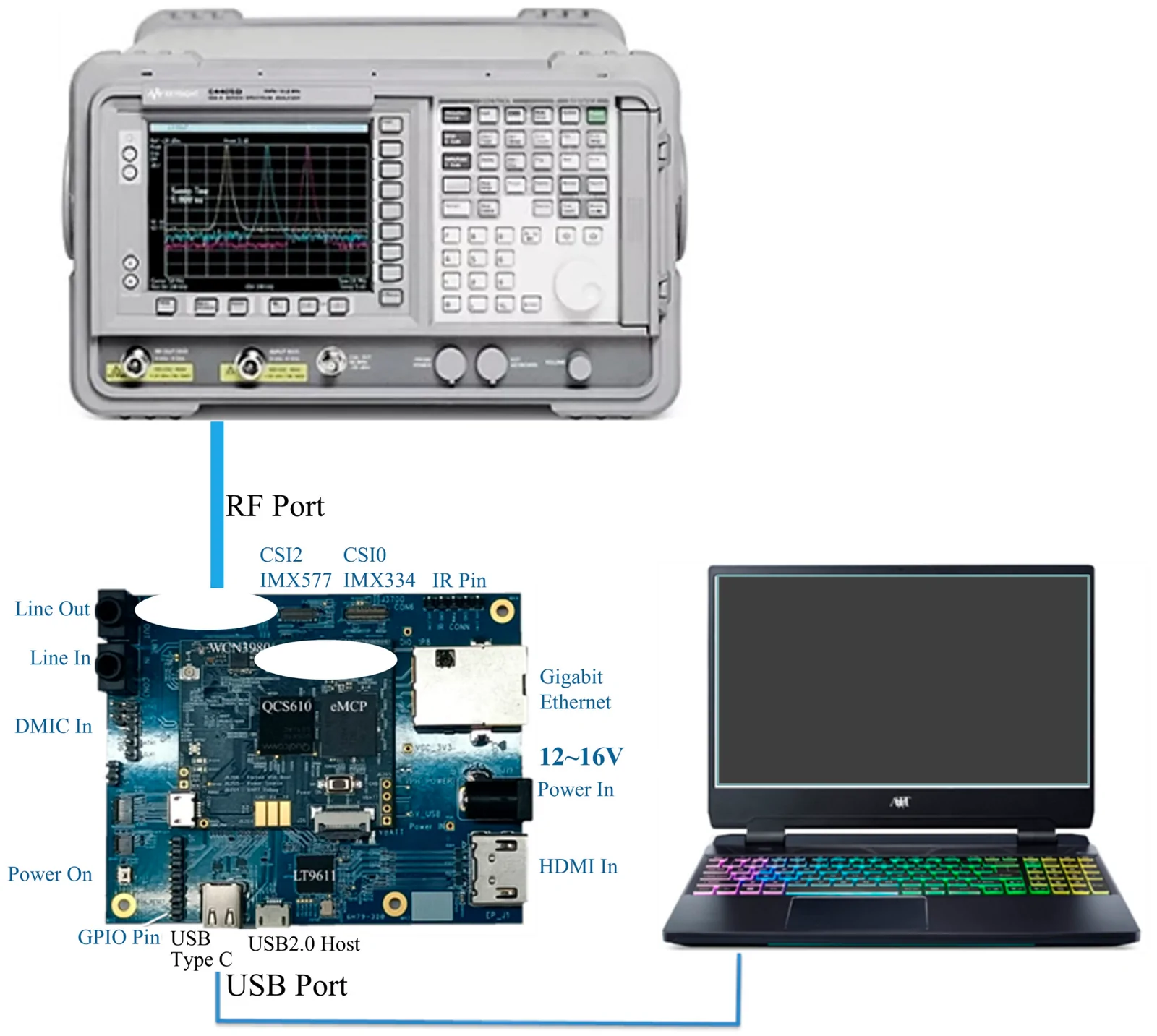
Spectrum analyzer setup.

A spectrum analyzer was used to examine the frequency-domain emission characteristics of the proposed embedded edge AI camera system. The measurement setup is shown in Figure 12.

The analysis aimed to characterize the system’s spectral behavior during operation and identify spurious emissions, harmonic components, and other undesired spectral signals. The measurement results provided a preliminary assessment of the system’s emission profile and served as a reference for the subsequent radiated emissions testing.

### 4.4. Radiated Emission Test Setup

**Figure 13:**
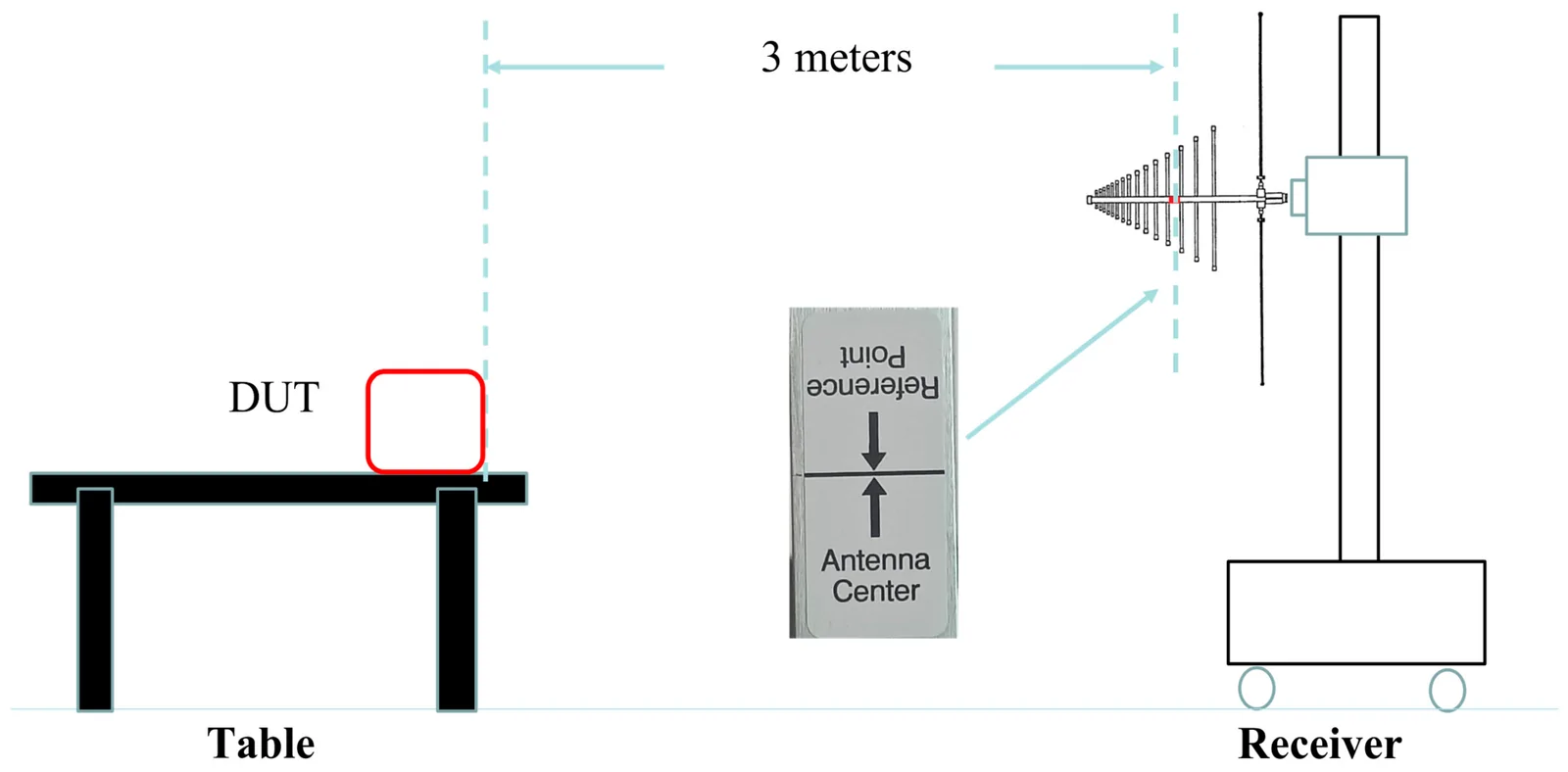
Radiated emission setup.

Radiated emissions testing was conducted to evaluate the electromagnetic emissions of the proposed embedded edge AI camera system under normal operating conditions. The test setup is illustrated in Figure 13.

During the test, the device under test (DUT) continuously performed its intended data-processing and wireless communication functions. Measurements were conducted at a distance of 3 m using both horizontal and vertical antenna polarizations. Peak and quasi-peak detection modes were applied over their respective frequency ranges. The measurements were intended to detect unintended radiation and harmonic components associated with the high-speed digital circuits, power circuitry, and wireless communication modules. The measured emission levels were compared with the applicable Class B limits to assess the electromagnetic compatibility (EMC) performance of the developed system.

### 4.5. Intelligent Recognition Evaluation Setup

**Figure 14:**
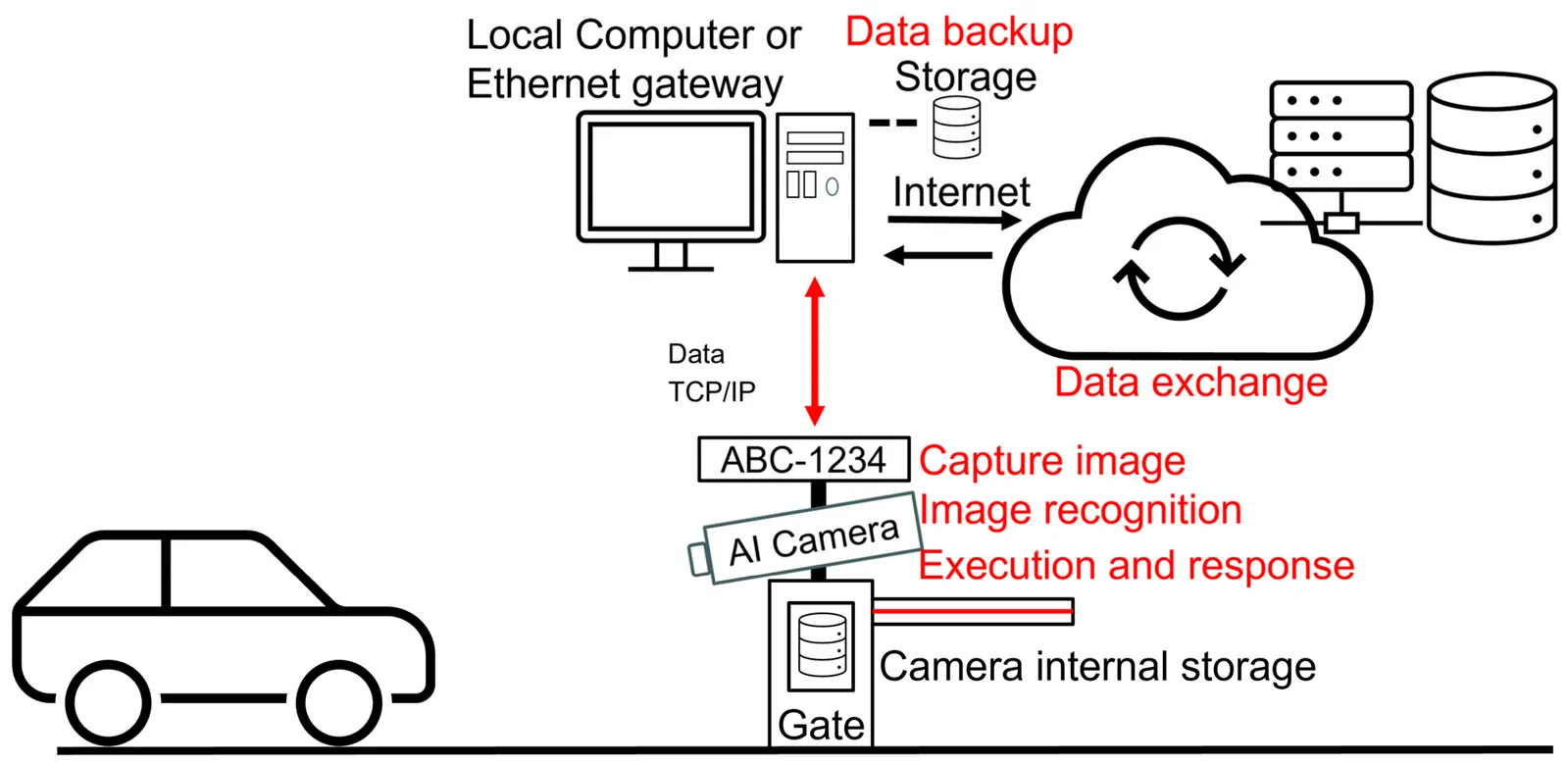
Parking lot application diagram.

To assess the practical applicability of the proposed embedded edge AI camera, we implemented a parking-management application that combines vehicle type detection and license plate localization. and license plate recognition. Figure 14 illustrates the application framework.

The edge device acquires images, performs inference, and generates vehicle type and license plate results locally. These results are stored on the device and can be transmitted to a central management system for monitoring, data logging, statistical analysis, and access control. They can also support automated parking-gate operation. The parking lot application serves as a case study of the system’s edge inference capability. Accordingly, we used existing pretrained models for vehicle type detection and plate text recognition, while fine-tuning a pretrained detector for license plate localization.

System performance was evaluated in terms of recognition accuracy and processing throughput. The following performance metrics were considered:

- Recognition accuracy (%) for vehicle type detection and license plate recognition.
- Processing throughput, expressed in frames per second (FPS), under multi-task and single-task operating conditions.

Tests were conducted under both multi-task and single-task operating conditions. In multi-task mode, the system performed four recognition operations per frame. A single-task configuration dedicated exclusively to license plate recognition was also evaluated to determine the maximum inference throughput of the proposed platform.

#### 4.5.1. AI Recognition Architecture and Model Deployment

The parking application uses three sequential model stages: vehicle type detection, license plate localization, and plate text recognition. Table 4. Model specifications and deployment settings for the three recognition stages, summarizing the model origin, deployed model file, architecture, input dimensions, output classes, parameters, MACs per call, calls/image, model file size, model precision and conversion, and Inference backend. The terms Stage 1–3 refer to the inference pipeline. The vehicle hardware evaluation phases described in Section 4.5.3 are separate tests of the application.

In Stage 1, for vehicle type detection, the system runs the pretrained SSD-MobileNetV2 detector (ssd_mobilenet_v2_quantized_coco.dlc) once on each input image. We directly used the pretrained ssd_mobilenet_v2_quantized_coco model released by the TensorFlow 1 Detection Model Zoo without retraining its weights. The source model was trained on COCO train2017 and evaluated on COCO val2017. This SSD-MobileNetV2 model accepts a 300 × 300 × 3 image and has approximately 6.06 million parameters and 0.77 billion multiply–accumulate operations (MACs) per call. The deployed model file, ssd_mobilenet_v2_quantized_coco.dlc, is approximately 24.4 MB. Post-processing retains only the car, bus, and truck categories required for the parking application. These three vehicle types were selected for the demonstrated parking-management application because vehicle-type information can be used to determine different parking fee categories. A single image may contain multiple vehicles, and the detector returns a separate bounding box and category for each detected vehicle. For deployment, the 32-bit floating-point (FP32) model representation was converted to an 8-bit integer (INT8) Deep Learning Container (DLC) for inference through the Snapdragon Neural Processing Engine (SNPE) on the QCS610 digital signal processor (DSP).

In Stage 2, for license plate localization, the system runs the SSD-MobileNetV2 detector (ssd_mobilenet_v2_plate.dlc) once for each vehicle detected in Stage 1. The detector was initialized with COCO-pretrained weights and fine-tuned for the single license plate class using the Car License Plate Detection dataset by andrewmvd on Kaggle. This dataset contains 433 images with license plate bounding boxes annotated in PASCAL VOC XML format. The images were assigned to training and validation at an expected ratio of approximately 8:2. The detector accepts a 300 × 300 × 3 image and has approximately 4.60 million parameters and 0.64 billion MACs per call. The deployed model file is approximately 18.7 MB. For deployment, the FP32 model representation was converted to an INT8 DLC for inference through SNPE on the QCS610 DSP (Qualcomm Technologies, Inc., San Diego, CA, USA). The resulting bounding box defines the plate region passed to Stage 3.

In Stage 3, for license plate character recognition, we directly used clovaai’s pretrained PyTorch model, None-ResNet-None-CTC.pth, associated with the scene text recognition framework of Baek et al. [57], without retraining or fine-tuning it on license plate data. The model uses a ResNet feature extractor and connectionist temporal classification (CTC) to recognize the complete plate character sequence without segmenting individual characters. It has no recurrent sequence-modeling layer. Each localized license plate image is converted to grayscale and resized to 32 × 100 × 1 pixels. The model has 37 output classes, comprising 26 letters, 10 digits, and one CTC blank class, with approximately 44.27 million parameters and 5.05 billion MACs per call.

For deployment, we exported the PyTorch model to Open Neural Network Exchange (ONNX), an open format for transferring models between deep learning frameworks, using opset 11. Output consistency between the PyTorch and ONNX models was verified. The ONNX model was then converted to a TensorFlow SavedModel using onnx-tf, followed by conversion to the float16 TensorFlow Lite model crnn_float16.tflite. The deployed file is approximately 88.6 MB. Its weights are stored in FP16 format, while inference is performed in FP32 precision on the QCS610 CPU.

As documented by Baek et al. [57] and the accompanying clovaai repository, the source framework uses MJSynth (MJ; approximately 8.9 million synthetic word images) and SynthText (ST; approximately 5.5 million cropped synthetic word images) for training. Its validation data comprise the union of the training partitions of IC13, IC15, IIIT, and SVT. The separate benchmark evaluation sets comprise IIIT, SVT, IC03, IC13, IC15, SVTP, and CUTE. These datasets describe the official pretrained model; the present study did not repeat its training, validation, or public benchmark evaluations.

#### 4.5.2. Dataset, Training, and Validation Configuration

The datasets and training configurations were selected according to the specific functions of the three-stage recognition pipeline: Stage 1, vehicle type detection; Stage 2, license-plate localization; and Stage 3, license-plate text recognition.

For Stage 1 (vehicle type detection), we adopted the pretrained ssd_mobilenet_v2_quantized_coco model from the TensorFlow 1 Detection Model Zoo. The source model was trained on COCO train2017 and evaluated on COCO val2017. In the present study, we used the pretrained weights directly, without retraining the model or conducting a new COCO validation. For the parking application, post-processing retained only detections labeled as car, bus, or truck. These three categories describe the outputs used by our application; they do not imply that the source model was trained using only three categories.

For Stage 2 (license plate localization), we fine-tuned an SSD–MobileNetV2 detector initialized with COCO-pretrained weights using the Car License Plate Detection dataset provided by andrewmvd on Kaggle. The dataset contains 433 images and 471 annotated license plate bounding boxes in PASCAL VOC XML format. The images were randomly divided into training and validation subsets at an approximate 8:2 ratio. The plate localization model achieved a validation mean average precision (mAP) of approximately 0.96 at an intersection-over-union (IoU) threshold of 0.5 (mAP@0.5). This metric evaluates predicted plate bounding boxes against the validation annotations; it does not represent plate character recognition accuracy or performance in the independent parking-lot test.

For Stage 3 (license plate character recognition), we directly used clovaai’s pretrained PyTorch None-ResNet-None-CTC.pth model associated with the scene text recognition framework of Baek et al. [57]. According to the official documentation, MJSynth (MJ; approximately 8.9 million synthetic word images) and SynthText (ST; approximately 5.5 million cropped synthetic word images) were used for training. The validation data comprised the union of the training partitions of IC13, IC15, IIIT, and SVT. Separate benchmark evaluation datasets comprised IIIT, SVT, IC03, IC13, IC15, SVTP, and CUTE. These training, validation, and benchmark datasets describe the source model’s documented procedure. We did not retrain or validate the OCR model, or repeat the public benchmark evaluations, in this study. Its performance in the proposed parking application was assessed through the independent real-world tests reported in Section 5.5.

The parking-lot field-test images were acquired separately from actual vehicles using the developed AI camera. None of these images were used to fine-tune or validate the Stage 2 license plate localization model. The Stage 1 and Stage 3 models were used with pretrained weights and were not retrained using field-test images. Thus, the field test provides an independent evaluation of the deployed system under practical parking-lot operating conditions.

#### 4.5.3. Real-World Field-Test Protocol and Evaluation Metrics

The integrated recognition system was tested with actual vehicles in outdoor and indoor parking environments. The test included two buses and two trucks outdoors and nine cars indoors, for a total of 13 vehicles and 13 license plates. The field-test data were acquired separately from the data used to fine-tune and validate the Stage 2 plate localization model. Stage 1 and Stage 3 used pretrained weights and were not retrained using the field-test images.

The camera processed the vehicle images using the three recognition stages described in Section 4.5.1. Stage 1 detected vehicles and assigned a vehicle type of car, bus, or truck. Stage 2 localized the license plate associated with each detected vehicle, and Stage 3 generated the plate character sequence. For each tested vehicle, the recorded vehicle type and plate number were compared with the corresponding ground truth. A vehicle was counted as correctly recognized only when both its type and its complete plate number were correct. The vehicle-level integrated recognition accuracy was calculated as follows:

(10) $$\mathrm{Recognition}\;{\mathrm{Accuracy}}_{\mathrm{integrated}}\;(\%)=\frac{N_{\mathrm{both}\;\mathrm{correct}}}{N_{\mathrm{tested}}}\times 100\%$$

where *N_both correct_* is the number of tested vehicles for which the vehicle was detected, its actual type (car, bus, or truck) was assigned, and the complete predicted plate character sequence exactly matched the actual plate number. *N_tested_* is the total number of tested vehicles (13 in this study). An incorrect or missing vehicle type or plate number caused the vehicle to be counted as incorrect. Percentages displayed alongside individual model predictions were not treated as integrated recognition accuracy. The processing rate of the integrated system was reported separately in frames per second (FPS).

The outdoor and indoor settings represented practical parking conditions, including relatively low lighting indoors. Lighting conditions were not experimentally controlled. Dedicated evaluations under nighttime, strong-light, or backlit conditions were not conducted in this study; therefore, accuracy for those conditions is not reported. The individual vehicle outcomes and aggregate field-test results are presented in Section 5.5.

## 5. Results and Discussion

This section presents the experimental results for the proposed embedded edge AI camera system, including signal integrity verification, RF transmitter characterization, radiated emissions evaluation, and intelligent recognition performance assessment. For signal integrity verification, the measured results are compared with the corresponding simulation results to evaluate the effectiveness of the PCB design. The RF, radiated emissions, and intelligent recognition results are used to assess the overall performance and practical applicability of the developed system.

### 5.1. Signal Integrity Verification Results

Following PCB fabrication and assembly, functional verification and electrical performance tests were conducted to confirm the proper operation of the developed hardware platform. The signal integrity of the high-speed interfaces was subsequently evaluated using a vector network analyzer (VNA).

The MIPI-DSI interface was selected as a representative high-speed differential link for signal integrity verification. Its measured S-parameter responses were compared with the corresponding simulation results, as summarized in Table 5. The measured and simulated responses exhibited consistent trends, and both satisfied the specified design requirements over the frequency range of interest.

The measured reflection coefficient (S_11_) and transmission coefficient (S_21_) parameters [47,58] were consistent with the simulated responses. The S_11_ results indicate limited signal reflection, while the S_21_ results demonstrate acceptable transmission loss across the evaluated frequency range. These results confirm that the implemented PCB stack-up and impedance-controlled routing provide satisfactory signal integrity for the MIPI-DSI differential interface.

### 5.2. RF Verification Results

The conducted RF transmitter performance of the proposed embedded edge AI camera system was evaluated using a LitePoint IQxel-MW wireless connectivity test system. The measurements characterized the integrated Wi-Fi and Bluetooth transmitters under normal operating conditions.

#### 5.2.1. Wi-Fi RF Transmitter Verification

**Figure 15:**
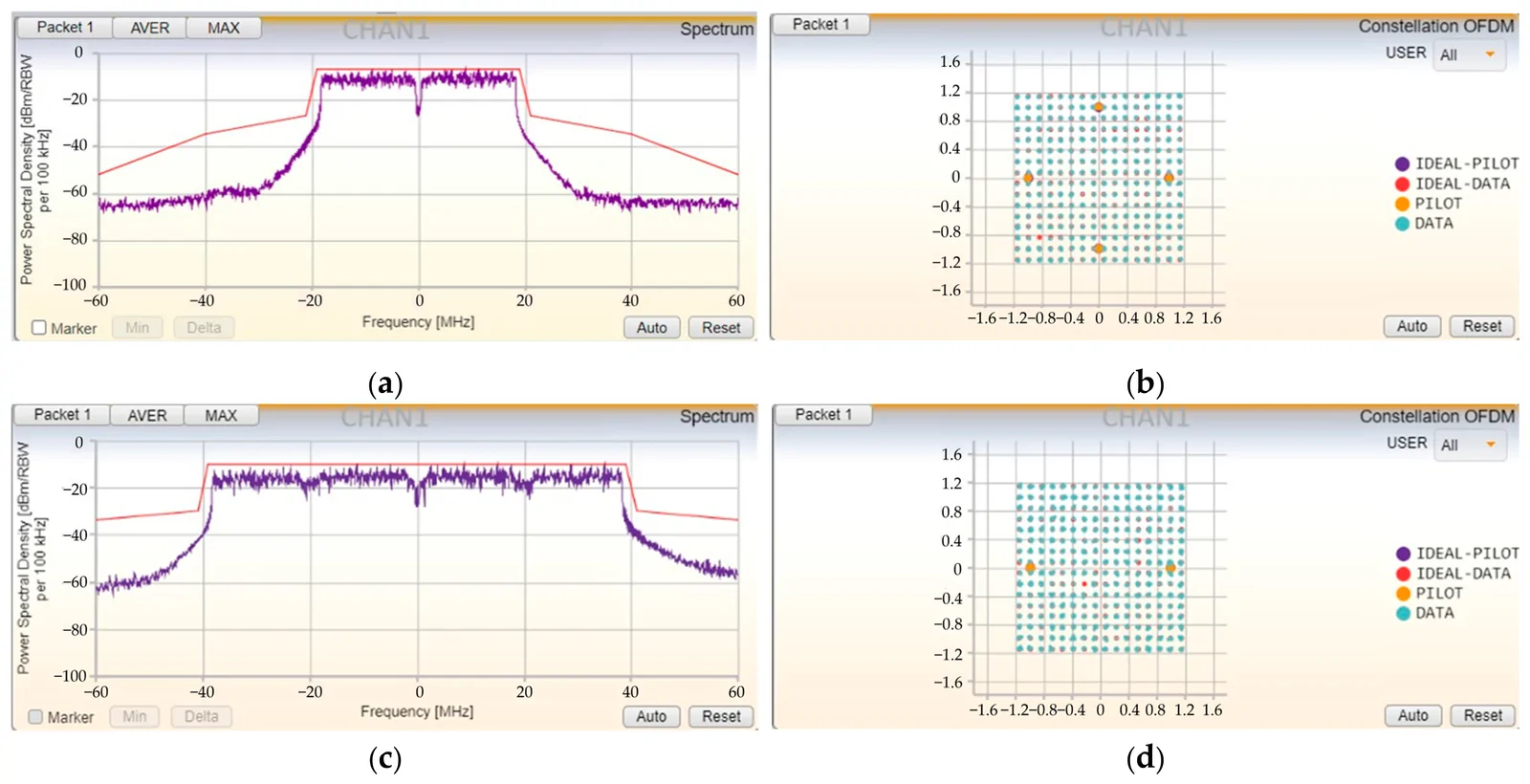
Wi-Fi OFDM transmitter measurement results obtained using the LitePoint IQxel-MW: (**a**) 2.4 GHz transmit spectral mask; (**b**) 2.4 GHz constellation diagram; (**c**) 5 GHz transmit spectral mask; and (**d**) 5 GHz constellation diagram. In (**a**) and (**c**), the purple traces represent the measured power spectra, while the red traces indicate the spectral mask limits.

The Wi-Fi orthogonal frequency-division multiplexing (OFDM) transmitter performance was evaluated in the 2.4 and 5 GHz bands. The measured error vector magnitude (EVM) values were −39.86 and −36.57 dB for the 2.4 and 5 GHz test configurations, respectively. As shown in Figure 15, the measured constellation points were closely clustered around their ideal symbol positions, indicating satisfactory OFDM modulation quality. In addition, the measured spectra remained within the applicable spectral-mask limits. The key measurement results, including average transmit power, frequency error, symbol-clock error, and EVM, are summarized in Table 6.

#### 5.2.2. Bluetooth RF Transmitter Verification

**Figure 16:**
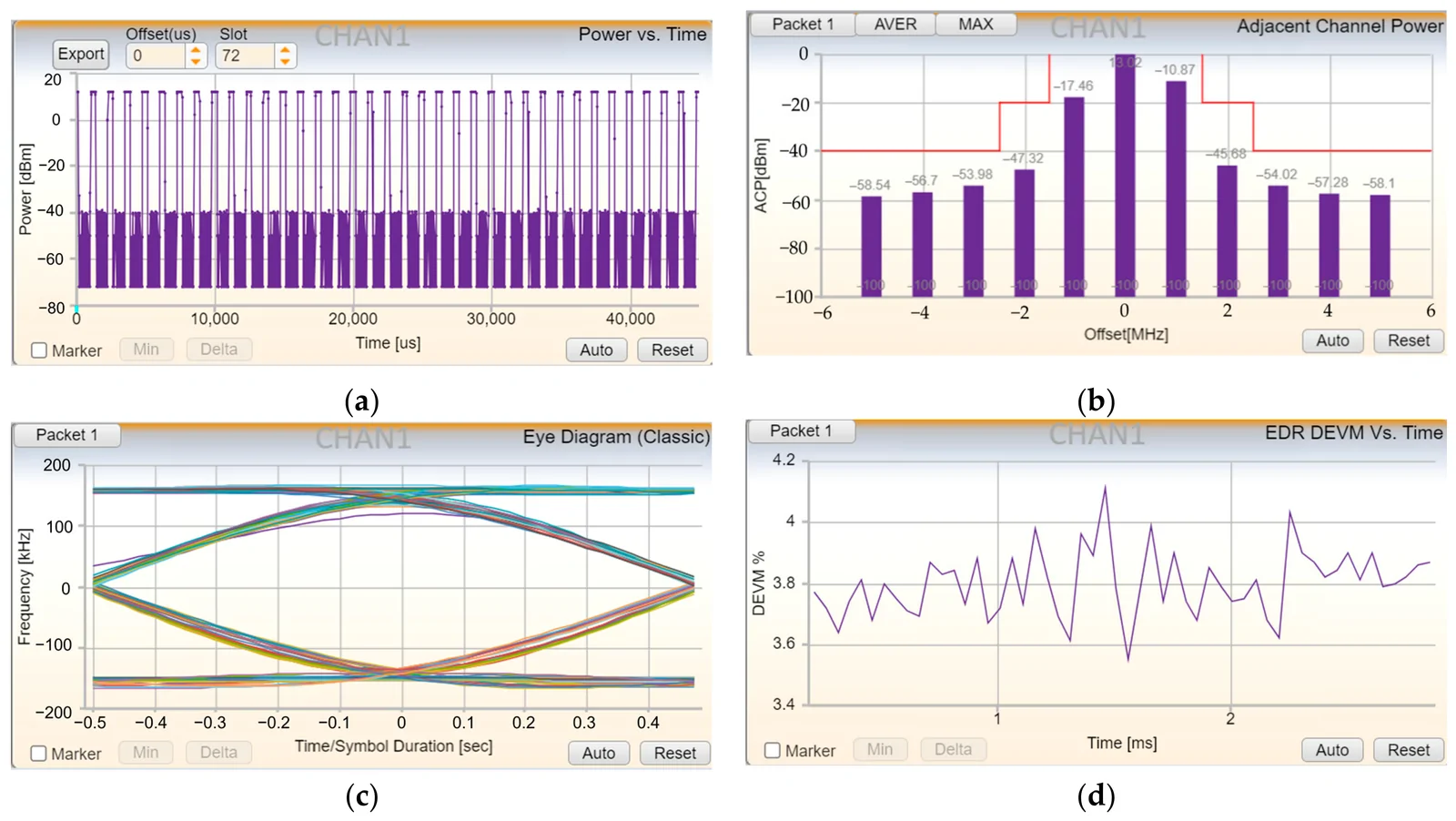
Bluetooth conducted RF test results: (**a**) transmit power versus time at 2440 MHz; (**b**) adjacent-channel power at 2440 MHz; (**c**) GFSK eye diagram for Bluetooth Classic Basic Rate at 2440 MHz; and (**d**) DEVM versus time for Bluetooth EDR at 2402 MHz.

The RF performance of the Bluetooth transmitter was evaluated at selected frequencies within the 2.4 GHz band using the LitePoint IQxel-MW wireless connectivity test system. As shown in Figure 16a, the transmit-power-versus-time response at 2440 MHz exhibited repeated transmission bursts with consistent power levels, indicating stable packet-based transmission. The adjacent-channel power measurement at 2440 MHz, presented in Figure 16b, was used to assess unwanted emissions in neighboring channels. The Bluetooth Classic Basic Rate (BR) Gaussian Frequency-Shift Keying (GFSK) eye diagram in Figure 16c exhibits a clearly defined eye opening, indicating consistent symbol transitions and satisfactory modulation quality. For the Enhanced Data Rate (EDR) measurement at 2402 MHz, the differential error vector magnitude (DEVM) ranged from 3.58% to 4.10% over the observed packet interval, as shown in Figure 16d. These results characterize the temporal power response, adjacent-channel emissions, GFSK modulation quality, and EDR modulation accuracy of the Bluetooth transmitter under the evaluated configurations.

Overall, the conducted RF measurements confirm that the Wi-Fi and Bluetooth transmitters satisfy the corresponding performance requirements under the evaluated operating conditions, supporting the suitability of the implemented RF circuitry and PCB layout for wireless communication in the developed embedded edge AI camera system.

### 5.3. Spectrum Analysis Results

**Figure 17:**
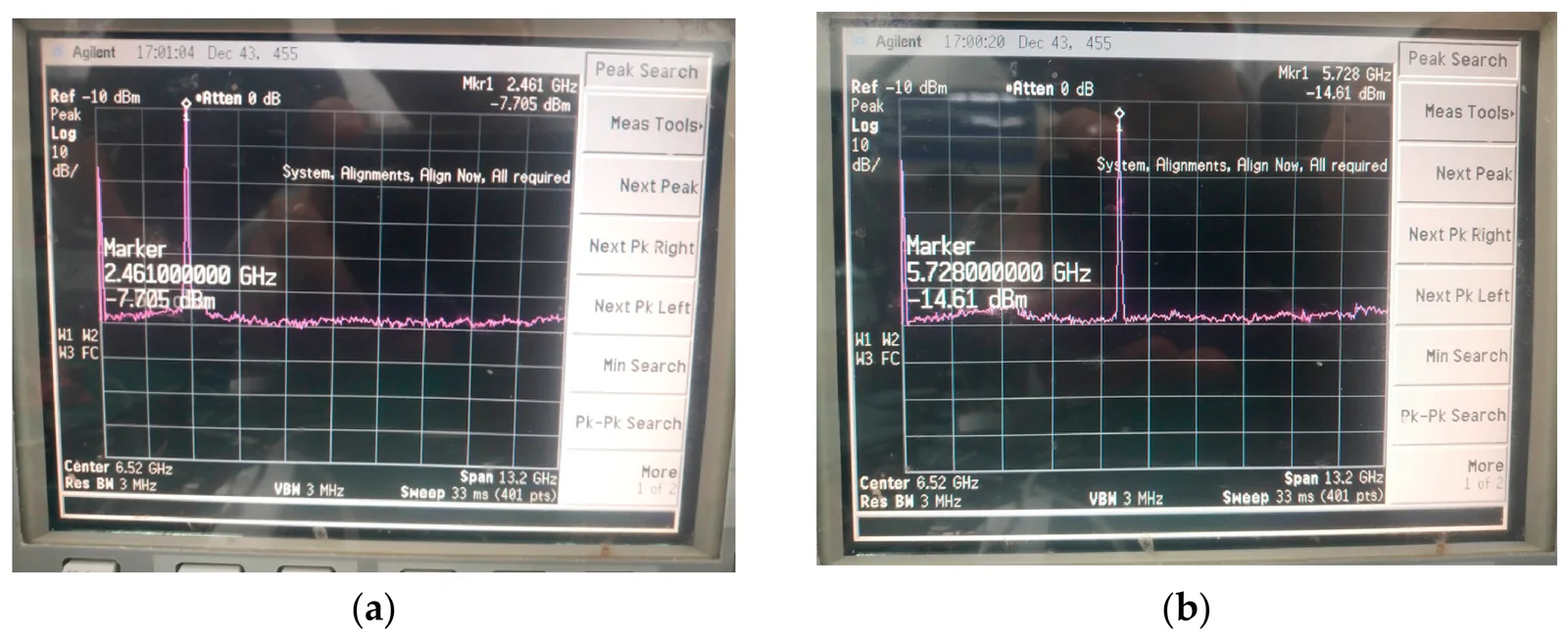
Conducted RF spectra of the Wi-Fi transmitter measured at: (**a**) 2.461 GHz and (**b**) 5.728 GHz.

An Agilent spectrum analyzer was used to characterize the Wi-Fi transmitter spectrum and examine potential harmonic and spurious emissions. As shown in Figure 17, distinct spectral components centered at 2.461 and 5.728 GHz were observed, with corresponding marker levels of −7.705 and −14.61 dBm, respectively. No prominent second- or third-order harmonic components were detected above the displayed noise floor under the evaluated conditions. While OFDM spectral-mask compliance and modulation quality were evaluated using the LitePoint IQxel-MW system in Section 4.2, the wide-span spectrum measurements presented here provide a complementary assessment of signal generation at the selected operating frequencies and the observable harmonic and spurious spectral components.

### 5.4. Radiated Emission Results

The radiated emissions of the proposed embedded edge AI camera system were evaluated in a semi-anechoic chamber under normal operating conditions. During testing, the device under test (DUT) continuously performed its intended wireless communication and data-processing functions. Measurements were conducted at a separation distance of 3 m using both horizontal and vertical antenna polarizations.

**Figure 18:**
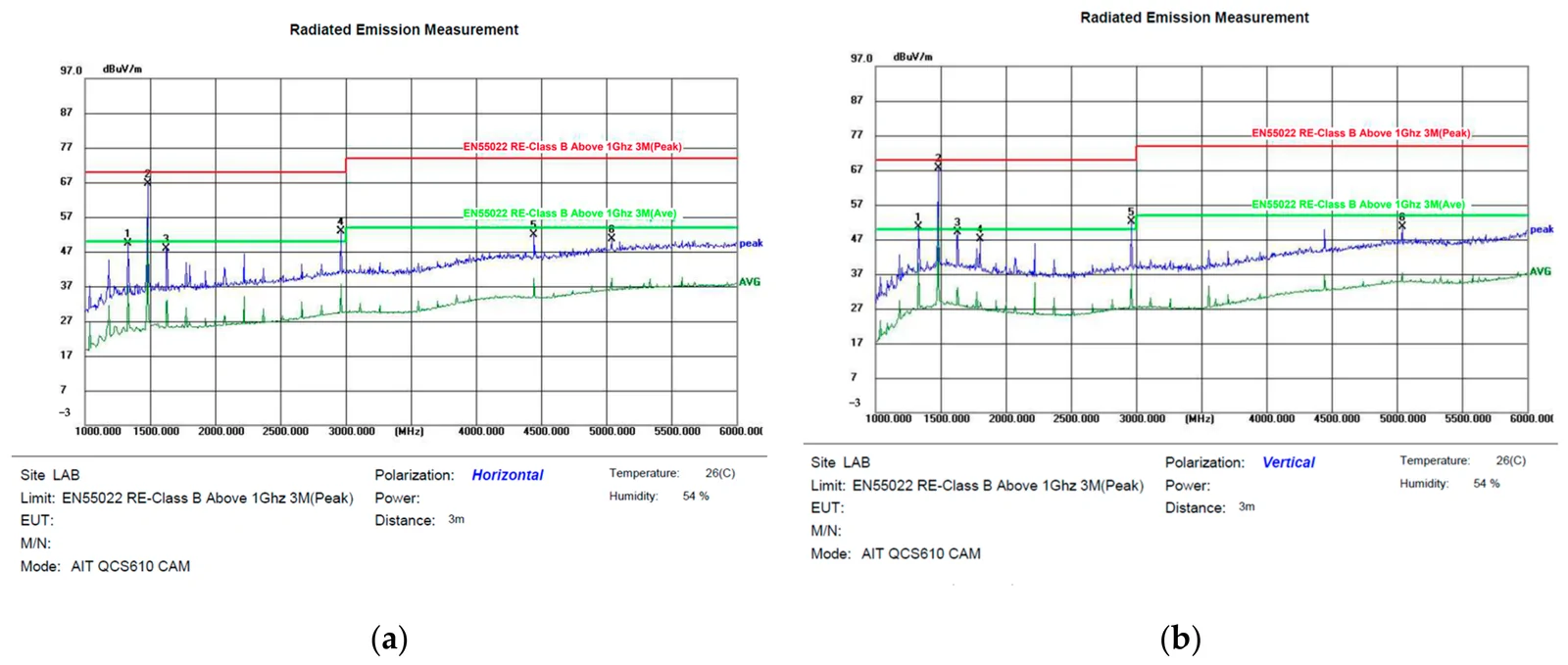

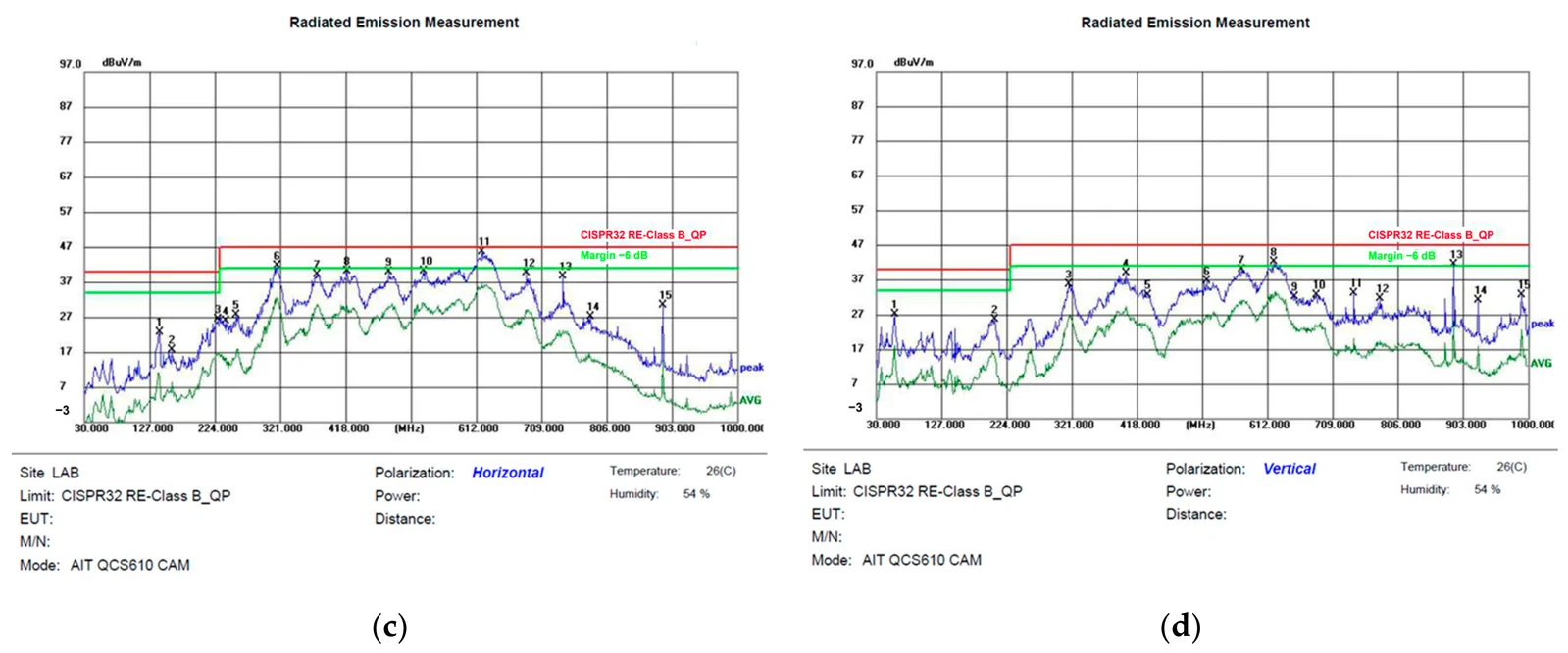
Radiated emission test results: (**a**) EN 55022 Class B above 1 GHz at 3 m, horizontal polarization (peak); (**b**) EN 55022 Class B above 1 GHz at 3 m, vertical polarization (peak); (**c**) CISPR 32 Class B, at 3 m, horizontal polarization (quasi-peak); and (**d**) CISPR 32 Class B, at 3 m, vertical polarization (quasi-peak).

Figure 18 presents the radiated emissions measurement results for the developed system. The measured emission levels were evaluated against the EN 55022 Class B and CISPR 32 Class B limits specified in the laboratory test report. All measured emissions remained below the applicable limits throughout the evaluated frequency range, confirming that the developed hardware platform satisfies the specified Class B radiated emissions requirements during wireless communication and data-processing operation.

### 5.5. Intelligent Recognition Results

**Figure 19:**
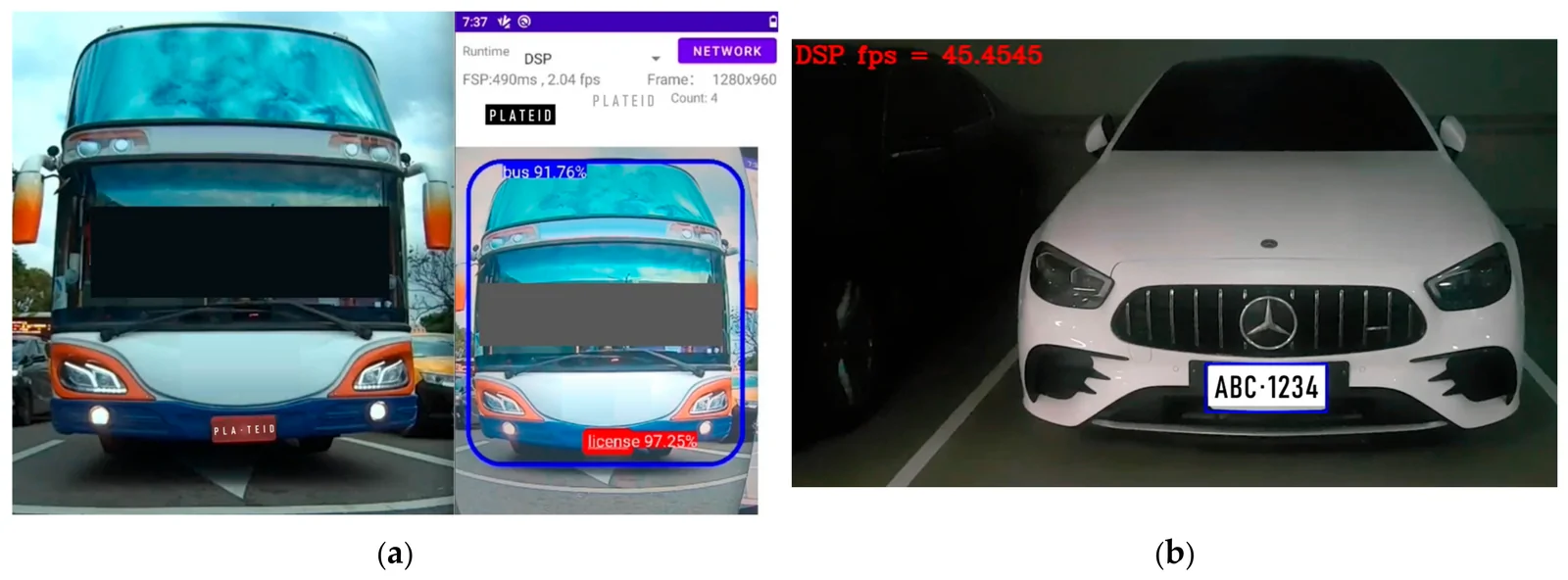
Representative vehicle type detection and license plate recognition output from the parking-lot field test: (**a**) a representative bus test result, where the displayed percentages (truck: 91.76%; license: 97.25%) represent confidence scores for the individual predictions; (**b**) a representative car test result showing a processing rate of approximately 45 FPS in single-task mode.

The proposed embedded edge AI camera was tested with 13 actual vehicles in outdoor and indoor parking environments. The sample comprised 2 buses and 2 trucks outdoors and 9 cars indoors. For every vehicle, the predicted vehicle type and complete license plate sequence were compared with the corresponding ground truth according to the integrated recognition criterion defined in Section 4.5.3. Figure 19 shows a representative system output, and Table 7 lists the results for each vehicle.

In Figure 19, the interface displays “truck 91.76%” and “license 97.25%” alongside the individual predictions. These percentages are confidence scores displayed for that image. They should not be interpreted as OCR accuracy or as the integrated recognition accuracy calculated from the 13-vehicle field test. The latter was determined by comparing the recorded vehicle type and complete plate sequence with the ground truth for each vehicle.

As shown in Table 8, both outputs were correct for all four outdoor vehicles: two buses and two trucks. The recorded outdoor conditions included two sunny and two cloudy observations. Both outputs were also correct for all nine indoor cars, comprising four observations recorded as normal light and five recorded as low light. Across the entire sample, 12 of 13 vehicles met the joint criterion: each was assigned the correct vehicle type and its complete plate sequence matched the ground truth. Equation (10) therefore yields a vehicle-level integrated recognition accuracy of 92.3% (12/13) for the tested vehicles. Among the 13 vehicles evaluated in the license plate recognition test, 12 license plates were correctly recognized, while 1 recognition error occurred. The error was mainly associated with the visual similarity between the characters “1” and “I,” which can be difficult to distinguish depending on the character font. Recognition performance may also be affected by weak or non-uniform illumination. Therefore, for parking-lot applications, adequate and reasonably uniform lighting is recommended in the entrance recognition area to facilitate reliable license plate recognition. Although the present study did not conduct extensive validation covering all possible environmental and illumination conditions, the experimental results indicate that the proposed system can meet the recognition requirements of the intended parking-lot application under appropriately controlled operating conditions.

The integrated system operated at approximately 2 frames per second (FPS) during the parking-lot test. This processing rate describes the complete vehicle type, plate localization, and character recognition pipeline; it is separate from the displayed prediction confidence scores and from the vehicle-level accuracy. The results demonstrate correct integrated outputs for the vehicles and lighting conditions recorded in this field test. The sample was limited to 13 vehicles, and the lighting categories were observational rather than controlled experimental conditions. Accordingly, the result describes this sample and should not be interpreted as an accuracy estimate for nighttime, strong-light, or backlit settings, which were not separately tested.

In a separate single-task test dedicated to license plate recognition, the system reached up to 45 FPS, compared with approximately 2 FPS for the integrated vehicle type and license plate recognition pipeline. These rates describe different task configurations and should not be interpreted as a direct comparison of the recognition models. Because inference is performed on the device, recognition results can be stored locally or transmitted to a central management system without transmitting every raw image for remote recognition. Network latency and bandwidth usage were not measured in this study.

The prototype’s DC input power was measured using a bench DC power supply set to 12 V. Representative current readings in the recorded test were approximately 0.183–0.527 A, corresponding to input powers of approximately 2.20–6.32 W. These readings refer to the powered prototype under the recorded test conditions rather than the QCS610 chip alone.

When the results were grouped according to the observed lighting conditions, the exact license-plate recognition rates were 2/2 (100%) under cloudy outdoor conditions, 2/2 (100%) under sunny outdoor conditions, 4/4 (100%) under indoor normal-light conditions, and 4/5 (80%) under indoor low-light conditions. The vehicle type was correctly recognized for all 13 vehicles. However, because of the small and unequal number of vehicles evaluated under each lighting condition, these results should be interpreted as descriptive observations of the field evaluation rather than as statistically representative scenario-specific performance.

The field evaluation involved a small set of vehicles and license plates, with unequal frame counts per vehicle. Controlled evaluations under nighttime, strong illumination, and backlighting conditions were not conducted. The integrated pipeline operated at approximately 2 FPS in the demonstrated parking application; its suitability for high-speed driving remains unverified. The reported single-task throughput represents a separate operating configuration. Cross-platform comparisons are limited by differences in workloads and measurement scope. Future work will include broader datasets, controlled illumination tests, and optimization of the integrated pipeline.

## 6. Conclusions

**Figure 20:**
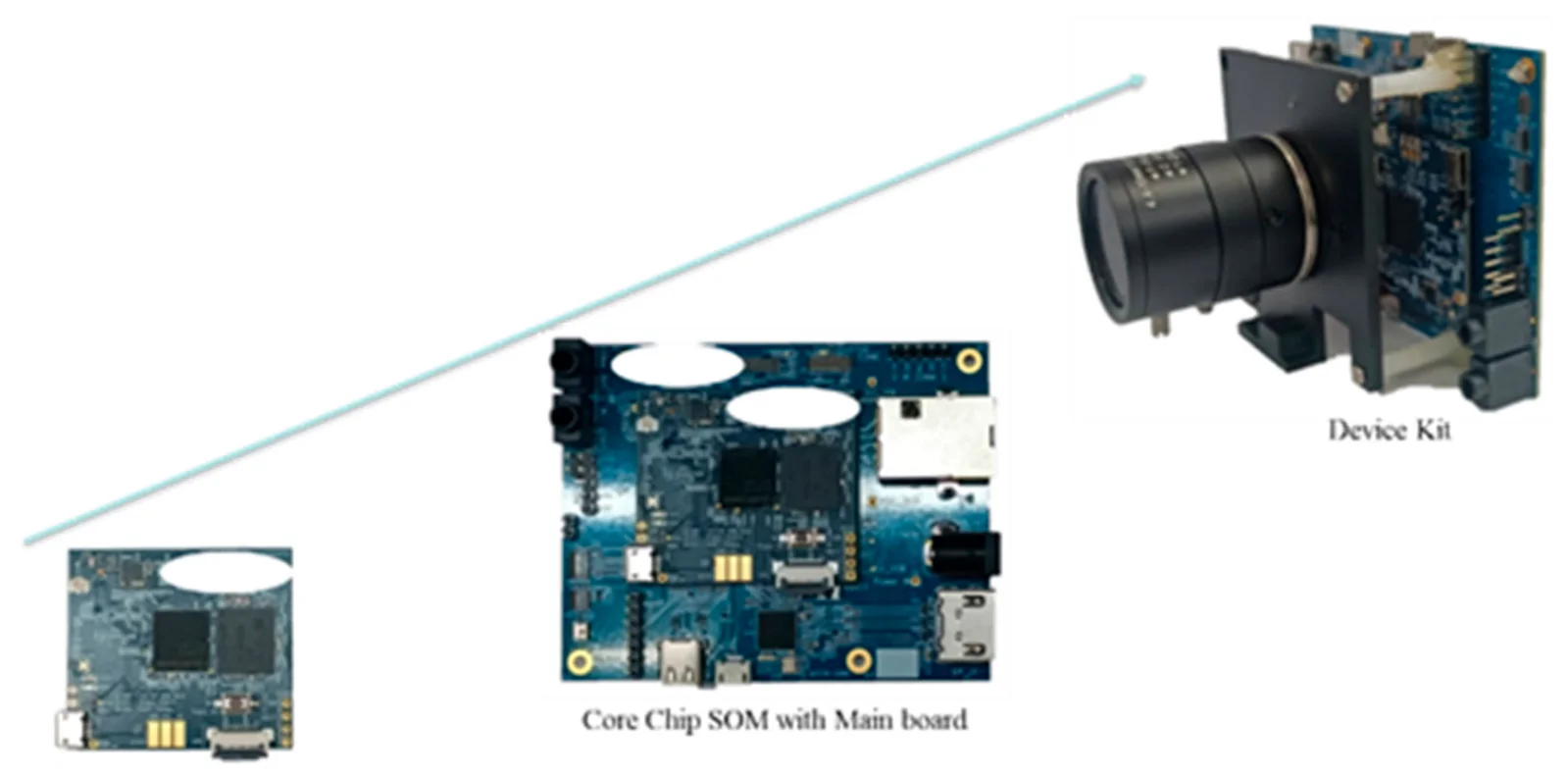
Product design stage.

This study presented the design and experimental validation of an embedded edge artificial intelligence camera system capable of image acquisition, intelligent recognition, real-time response, and event reporting. Figure 20 shows the completed system. The hardware development encompassed circuit implementation, impedance-controlled PCB routing, high-speed signal layout, signal integrity verification, RF transmitter performance evaluation, spectrum analysis, and radiated emissions testing.

The simulated and measured signal integrity responses of the MIPI-DSI interface exhibited consistent trends and satisfied the specified design requirements, confirming the suitability of the implemented PCB stack-up and impedance-controlled routing. The Wi-Fi and Bluetooth transmitters also satisfied the corresponding RF performance requirements, while the measured radiated emissions remained below the applicable Class B limits. In a parking-lot field test using real vehicles, the system achieved vehicle type detection and license plate recognition confidence scores of 91.76% and 97.25%, respectively, while operating in multi-task mode at an image resolution of 1280 × 960 pixels and a processing rate of 2 FPS. For all 13 tested vehicles—two buses, two trucks, and nine cars—both the predicted vehicle type and the complete license plate number matched the ground truth, yielding an integrated recognition accuracy of 12/13 (92.3%). When configured exclusively for license plate recognition, the system achieved a processing throughput of up to 45 FPS, demonstrating its capability for high-speed, real-time edge inference.

The system employs an 11 nm edge computing processor and a highly integrated hardware architecture, supporting a compact implementation and flexible deployment across AI and edge computing applications. By performing AI inference and data processing directly on the device, the proposed architecture can reduce network bandwidth requirements, server workload, raw-data transmission, and response latency while maintaining reliable recognition performance.

The field test involved a limited number of vehicles under observed outdoor and indoor lighting conditions; nighttime, strong-light, and backlit conditions were not separately evaluated. Further testing with more vehicles and controlled lighting conditions is needed to characterize performance beyond the scenarios examined here.

Overall, the results demonstrate the practical feasibility of integrating AI inference, wireless communication, and high-speed image processing into a compact embedded edge computing platform for intelligent parking management, intelligent transportation systems, and related edge vision applications.

## Figures and Tables

**Table 1:** Comparison of the proposed camera system with selected embedded vision platforms and published implementations.

Study/Platform	Task/Implementation	Reported Recognition Metric	Reported Throughput and Input	Power and Scope	Dimensions (mm) and Scope	Reported Cost and Basis
This study/QCS610	Vehicle type and plate recognition	Vehicle type: 91.76%; plate: 97.25%	~2 FPS integrated, input 1280 × 960; up to 45 FPS single-task	~2.20–6.32 W (prototype DC input; observed in the recorded test)	SOM 45 × 40; carrier 88.3 × 70	NR (prototype hardware cost)
TI Jacinto TDA4VM [6,7,8,9]	YOLOX-S-Lite detection (SK-TDA4VM)	AP: 38.3% (COCO 2017 val; model benchmark)	~30 FPS (YOLOX-S-Lite; input: 640 × 640)	NR for a comparable workload	24 × 24 (IC package)	NR (IC cost)
Toshiba TMPV7506XBG [10,11]	HOG-based pedestrian detection/ADAS	NR	FPS: NR; supports up to 1.3-MP cameras	~0.74 W average (processor; concurrent applications)	27 × 27, IC package	JPY 6000; IC sample, tax incl.; 2011
Toshiba TMPV7608XBG [12,13]	Enhanced CoHOG/SfM automotive vision	NR	Concurrent ADAS processing within 50 ms; supports Full-HD cameras	~3.4 W (MPEs + image-processing accelerators)	27 × 27, IC package	JPY 10,000; IC sample, tax incl.; 2014
Jetson Nano [2,14,15,16]	Three-model ADAS pipeline	Lane model: accuracy 66.02%; IoU 0.6363	15 FPS at detector input 224 × 224; 11 FPS at 640 × 480	5/10 W module reference profiles; not measured ADAS power	69.6 × 45, Nano 4GB module	USD 129; module launch, ≥1000 units; 2019
Jetson TX2 NX [2,17]	Three-model ADAS pipeline	Lane model: accuracy 66.02%; IoU 0.6363	27 FPS at detector input 224 × 224; 23 FPS at 640 × 480	7.5/15 W power modes; application power NR	~70 × 45 (TX2 NX module)	USD 149 (2021 module launch price; volume pricing)
Jetson TX2 8GB [18,19,20]	Embedded AI module; Pascal GPU	NR for the selected application	NR for the selected application	7.5/15 W module reference profiles	87 × 50, module	USD 399; module launch, ≥1000 units; 2017
AGX Xavier [20,21]	Radar/video fusion; INT8 YOLOv3, 2 ROIs	Overall P 73.1%; R 62.2%; mAP 54.1%; car AP 93.5%	17.39 FPS average system rate; model input 416 × 416 × N	10/15/30 W module reference profiles; not measured fusion power	100 × 87, module	USD 1099 module launch, ≥1000 units; 2018

NR = not reported, or not available. P = precision; R = recall; AP = average precision; N = number of model-input channels.

**Table 2:** PCB impedance requirements.

Interface	SE/Diff (Ω)	Impedance Requirement (Ω)
SPMI	SE	50 ± 10
RMII	SE	50 ± 10
WLAN IQ	Diff Z_0_	85 ± 15
Ethernet/HDMI	Diff Z_0_	100 ± 5
USB3.1	Diff Z_0_	90 ± 9
USB2.0	Diff Z_0_	90 ± 9
MIPI-DPHY	Diff Z_0_	85 ± 15
	SE	44 ± 8

**Table 3:** Impedance simulation results.

Parameters	Outer				Inner (Offset Stripline)			
	Single-Ended	Differential Signals			Single-Ended	Differential Signals		
		USB	MIPI	Ethernet/ HDMI		USB	MIPI	Ethernet/ HDMI
H1, Substrate 1 Height (mil)	3.09	3.09	3.09	3.09	34.10	34.10	34.10	34.10
Er1, Substrate 1 Dielectric	4.20	4.20	4.20	4.20	4.20	4.20	4.20	4.20
H2, Substrate 2 Height (mil)					4.24	4.24	4.24	4.24
Er2, Substrate 2 Dielectric					3.69	3.69	3.69	3.69
W1, Lower Trace Width (mil)	4.00	4.50	5.00	4.00	4.00	3.50	4.50	3.03
W2, Upper Trace Width (mil)	3.40	3.90	4.40	3.40	3.50	3.00	4.00	2.53
S1, Trace Separation (mil)		5.0	5.0	6.38		5.16	7.00	6.81
T1, Trace Thickness (mil)	1.40	1.40	1.40	1.40	1.20	1.20	1.20	1.20
C1, Coating Above Substrate (mil)	1.70	1.70	1.70	1.70				
C2, Coating Above Trace (mil)	0.60	0.60	0.60	0.60				
C3, Coating Between Traces (mil)		1.70	1.70	1.70				
CEr, Coating Dielectric	3.80	3.80	3.80	3.80				
Z_0_, Impedance (Ω)	55.04				50.34			
SPEC of Z_0_ (Ω)	50 ± 10				50 ± 10			
Pass/Fail	PASS				PASS			
Zdiff, Differential Impedance (Ω)		89.15	85.35	97.79		91.77	85.86	100.75
SPEC of Zdiff, (Ω)		90 ± 9	85 ± 15	100 ± 5		90 ± 9	85 ± 15	100 ± 5
Pass/Fail		PASS	PASS	PASS		PASS	PASS	PASS

**Table 4:** Model specifications and deployment settings for the three recognition stages.

Specification	Stage 1	Stage 2	Stage 3
Task	Vehicle type detection	License plate localization	License plate character recognition
Model origin	TensorFlow 1 Detection Model Zoo pretrained model; no retraining	COCO-pretrained SSD–MobileNetV2; fine-tuned for plate localization	clovaai pretrained model; no retraining
Deployed model file	ssd_mobilenet_v2_quantized_coco.dlc	ssd_mobilenet_v2_plate.dlc	crnn_float16.tflite
Architecture	SSD–MobileNetV2	SSD–MobileNetV2	2ResNet–CTC; no recurrent layer
Input	300 × 300 × 3	300 × 300 × 3	32 × 100 × 1
Output used	Car, bus, and truck, bounding boxes and categories	License plate bounding box	Complete plate character sequence; 37 output classes (26 letters, 10 digits, and one CTC blank class)
Parameters	≈6.06 million	≈4.60 million	≈44.27 million
MACs per call	≈0.77 billion	≈0.64 billion	≈5.05 billion
Calls/image	1 per input image (multiple vehicles may be detected in each image)	1 per detected vehicle	1 per localized plate
Model file size	≈24.4 MB	≈18.7 MBB	≈88.6 MB
Model precision and conversion	FP32 → INT8 DLC (quantization)	FP32 → INT8 DLC (quantization)	FP16 weight storage; FP32 execution
Inference backend	SNPE on QCS610 DSPS	SNPE on QCS610 DSP	TensorFlow Lite on QCS610 CPU

**Table 5:** Comparison of Simulated and Measured S11 and S21 Results.

S11 @450 MHz Target ≤ −15 dB	Simulation (dB)	Measured (dB)	Pass/Fail	S21 @450 MHz Target ≥ −2.0 dB	Simulation (dB)	Measured (dB)	Pass/Fail
DSI CLK	−18.3	−17.281	Pass	DSI CLK	−0.3	−1.0053	Pass
DSI Data0	−18.4	−17.158	Pass	DSI Data0	−0.3	−1.0411	Pass
DSI Data1	−19	−18.782	Pass	DSI Data1	−0.3	−1.0294	Pass
DSI Data2	−18.8	−16.973	Pass	DSI Data2	−0.3	−1.049	Pass
DSI Data3	−18.8	−16.032	Pass	DSI Data3	−0.3	−1.0353	Pass

**Table 6:** Wi-Fi OFDM transmitter performance in the 2.4 and 5 GHz bands.

Frequency Band	Signal Format	Channel BW (MHz)	Average TX Power (dBm)	Frequency Error (kHz)	Symbol-Clock Error (ppm)	EVM (dB)	Spectral-Mask Result
2.4 GHz	WLAN OFDM	40	15.46	−1.589	−0.60	−39.86	Pass
5 GHz	IEEE 802.11ac VHT OFDM	80	14.74	−3.041	−0.39	−36.57	Pass

**Table 7:** Model and license plate recognition summary.

Item	Content	Description
AI Inference	TensorFlow Lite (TFLite)	Qualcomm Snapdragon Neural Processing Engine (SNPE) on Digital Signal Processor
Place	Parking lot entrance	One car at a time
Item	Output Result	The vehicle type first, then the license plate The result will be output if the results of 4 consecutive frames (license plate) are the same.
	Vehicle Type	20 frames per second Identification confidence score: 91.76%
	License Plate	2 frames per second; output the result if 4 consecutive frames’ results are the same. Identification confidence score: 97.25% NOTE: Performance can reach up to 45 frames per second if only for license plate recognition.
Data Process	Control parking lot gates Process gate situation in time	Internal memory (eMMC and SD card) can be used for local storage. Vehicle information can be sent to the central control system for data backup, statistics, and other applications.

**Table 8:** Summary of the real-world evaluation results and test conditions.

No.	Setting	Lighting	Ground-Truth Vehicle Type	Ground-Truth Plate	Predicted Vehicle Type	OCR Result	* Max FPS	Time (s)	** Integrated Result
1	Outdoor	Cloudy	Bus	KAB0865	Bus	KAB0865	2.02	~3	Correct
2	Outdoor	Sunny	Truck	BAU9359	Truck	BAU9359	2.20	~2	Correct
3	Outdoor	Sunny	Bus	862TT	Bus	862TT	1.95	~2	Correct
4	Outdoor	Cloudy	Truck	8219EQ	Truck	8219EQ	1.90	~4	Correct
5	Indoor	Normal Light	Car	BJX6697	Car	BJX6697	2.04	~6	Correct
6	Indoor	Normal Light	Car	AJH6662	Car	AJH6662	2.07	~7	Correct
7	Indoor	Normal Light	Car	ATL9785	Car	ATL9785	2.06	~5	Correct
8	Indoor	Low Light	Car	ATE6903	Car	ATE6903	2.06	~8	Correct
9	Indoor	Low Light	Car	ALK9366	Car	ALK9366	1.96	~6	Correct
10	Indoor	Low Light	Car	BAU3966	Car	BAU3966	2.09	~5	Correct
11	Indoor	Low Light	Car	ANA1836	Car	ANA1836	2.12	~9	Correct
12	Indoor	Normal Light	Car	RDC2992	Car	RDC2992	2.14	~4	Correct
13	Indoor	Low Light	Car	AXG1806	Car	AXGI806	2.12	~9	Error
Total	4 Outdoor, 9 Indoor	2 Cloudy, 2 Sunny, 4 Normal Light, 5 Low Light	3 Types, 9 Cars, 2 Trucks, 2 Buses	13 Plates	Type: 13 Correct	Plates: 12 Correct, 1 Error	--	~70	12 Correct, 1 Error

* The processing rate varied from approximately 1.8 to 2.2 FPS during the tests. The FPS value reported for each vehicle represents the maximum value observed during that test. ** An integrated result was counted as correct only when both the predicted vehicle type and the complete OCR output matched the corresponding ground truth.

## Data Availability

All data supporting the findings of this study are available within the article.

## Abbreviations

The following abbreviations are used in this manuscript:

AC	Alternating Current
ADAS	Advanced Driver Assistance Systems
AI	Artificial Intelligence
ALPR	Automatic License Plate Recognition
BR	Basic Rate
B2B	Board-to-Board
CTC	Connectionist Temporal Classification
DEVM	Differential Error Vector Magnitude
DSP	Digital Signal Processor
DRC	Design Rule Check
DUT	Device Under Test
ECUs	Electronic Control Units
EDR	Enhanced Data Rate
EMC	Electro-Magnetic Compatibility
EMI	Electro-Magnetic Interference
EVM	Error Vector Magnitude
FP	Floating-Point
FPS	Frames Per Second
GFSK	Gaussian Frequency-Shift Keying
INT	Integer
IoU	Intersection-over-Union
ISP	Image Signal Processor
LRR	Long-Range Radar
MACs	Multiply–Accumulate Operations
MIPI-DSI	MIPI Display Serial Interface
mAP	mean Average Precision
OFDM	Orthogonal Frequency-Division Multiplexing
ONNX	Open Neural Network Exchange
PCB	Printed Circuit Board
PI	Power Integrity
PMIC	Power Management Integrated Circuits
RF	Radio Frequency
RSU	Roadside Unit
SI	Signal Integrity
SNPE	Snapdragon Neural Processing Engine
SoC	System-on-Chip
SPMI	System Power Management Interface
TFLite	TensorFlow Lite
TOPS	Tera-Operation Per Second
VNA	Vector Network Analyzer
VSWR	Voltage Standing Wave Ratio
